# Regulatory effects on virulence and phage susceptibility revealed by *sdiA* mutation in *Klebsiella pneumoniae*

**DOI:** 10.3389/fcimb.2025.1562402

**Published:** 2025-03-13

**Authors:** Sergio Silva-Bea, Pablo Maseda, Ana Otero, Manuel Romero

**Affiliations:** Department of Microbiology and Parasitology, Faculty of Biology - Aquatic One Health Research Center (ARCUS), Universidade de Santiago de Compostela, Santiago de Compostela, Spain

**Keywords:** quorum sensing, biofilm, AHL (N-acyl-homoserine lactone), serum resistance, bacteriophage, *Galleria mellonella*, SdiA

## Abstract

**Introduction:**

The World Health Organization has identified multi-drug resistant *Klebsiella pneumoniae* strains as the highest priority in 2024. Understanding the regulatory routes of virulence features is crucial for the development of novel anti-virulence strategies. SdiA, a LuxR-like quorum sensing (QS) receptor that responds to *N-*acyl-homoserine lactones (AHLs), is involved in the regulation of virulence traits in some Gram-negative bacteria. The function of this receptor in the virulence of *K. pneumoniae* remains uncertain. The objective of the present study was to elucidate the function of SdiA in *K. pneumoniae* biofilm formation and virulence.

**Methods:**

To this end, a genetic knockout of *sdiA* was conducted, and virulence-related phenotypic studies were performed following AHL provision.

**Results and Discussion:**

The results demonstrate that *sdiA* deficiency increases susceptibility to phage infection and human serum resistance, and promotes biofilm maturation and cell filamentation, although no effect on virulence was observed *in vivo* in the *Galleria mellonella* infection model. On the other hand, C6-HSL promoted *sdiA*-dependent biofilm maturation, capsule production and serum resistance while reducing virulence against *G. mellonella* in the absence of *sdiA*. The addition of C6-HSL did not affect phage susceptibility. The results of this study demonstrate that AHLs and SdiA exert a dual influence on virulence phenotypes, operating both independently and hierarchically. These findings provide new insights into the virulence of *K. pneumoniae* and its regulation by SdiA.

## 1 Introduction

The emergence of MDR bacteria represents a significant public health concern, given the lack of effective treatment options (Tacconelli et al., 2018). *K. pneumoniae* has been classified by the WHO as a maximum priority pathogen for the development of new antimicrobial strategies in 2024, with an increasing incidence of convergent strains with MDR and hypervirulence traits (González- et al., 2021; WHO, 2024).

A comprehensive understanding of the factors that regulate virulence traits is essential for the development of novel anti-virulence therapies. Among these, blocking QS systems may play an important role in controlling virulence in many MDR pathogens. QS systems are responsible for regulating gene expression in bacterial populations in accordance with cell density through the production of autoinducing molecules, which act as signals (Wang et al., 2020). In Gram-negative bacteria, these are AHLs, which are often synthesised by LuxI-type synthases and recognised by LuxR-type receptors (Papenfort and Bassler, 2016). In the case of some species of Enterobacteriaceae, such as *Escherichia coli*, *Salmonella* spp. and *Klebsiella pneumoniae*, a putative LuxI synthase is absent, yet an orphan LuxR receptor (SdiA) is present and it has been shown that this receptor is capable of detecting AHLs produced by other bacteria (Sabag-Daigle et al., 2015; Michael et al., 2001; Ahmer, 2004; Sabag-Daigle and Ahmer, 2012; Cao et al., 2022; Mayer et al., 2023), among other ligands (Janssens et al., 2007; Styles et al., 2020).

The regulatory role of SdiA in virulence has been widely investigated in *E. coli* and *Salmonella* (Ahmer, 2004; Mayer et al., 2023; Schwieters and Ahmer, 2024). In *E. coli*, it has been shown that SdiA has a promoting effect on survival in the gastrointestinal tract through acid tolerance upregulating *gad* expression (Sabag-Daigle et al., 2012), and the promotion of resistance to quinolones through expression of AcrAB efflux pump (Rahmati et al., 2002). However, these observations were made only in the context of *sdiA* being overexpressed, and no such phenotypes were observed when *sdiA* was in its native position on the chromosome (Dyszel et al., 2010b). Conversely, SdiA has also been demonstrated to exert a repressive effect on other phenotypes, including motility and adhesion, through repression of *fliC* (flagella) and *fimA* (fimbriae) expression (Mayer et al., 2023). Furthermore, SdiA has been proposed as a repressor of biofilm formation, as *sdiA*-lacking strains show higher biofilm forming ability through *uvrY* repression (Suzuki et al., 2002). In addition, a reduction in biofilm formation (Lee et al., 2009) and an increased phage sensitivity (Ghosh et al., 2009) has been observed in *E. coli* following the addition of AHL in an *sdiA*-dependent manner. Nevertheless, there are also studies that argue that SdiA does not affect biofilm formation in this species (Sabag-Daigle et al., 2012). However, the majority of laboratory strains of *E. coli* are low biofilm formers, which may result in an underestimation of the impact of QS on this phenotype (Król et al., 2019). Regarding *Salmonella*, SdiA has shown to regulate two loci in an AHL-dependent activation manner: *srgE* and *rck* (Smith and Ahmer, 2003; Schwieters and Ahmer, 2024). The first one, *srgE*, codifies an effector secreted by the type III secretor system involved in virulence (Habyarimana et al., 2014); and the second one, *rck*, is involved in adhesion to eukaryotic cells and resistance to complement killing in human serum (Ahmer et al., 1998; Michael et al., 2001; Mambu et al., 2017), suggesting a role of SdiA in pathogenesis. Another study has highlighted the significance of SdiA in *Salmonella* adhesion and biofilm formation, as adhesion to eukaryotic cells and biofilm formation was reduced in the mutant strain irrespective of the presence of AHL (Askoura et al., 2021). However, the contribution of SdiA to the pathogenesis of *Salmonella* remains unclear, as SdiA AHL-activated strains exhibit no greater advantage than *sdiA*-deficient strains in the gut environment (Smith et al., 2008; Dyszel et al., 2010a; Schwieters and Ahmer, 2024). Furthermore, *sdiA* expression levels in *Salmonella* can exhibit considerable fluctuations in biofilm cells, contingent on the culture medium and the duration of the incubation period (Wang et al., 2016).

The function of SdiA has also been the subject of investigation in the bacterium *Enterobacter cloacae*. Research has indicated that the inactivation of *sdiA* has a positive effect on biofilm formation and adhesion (Shankar et al., 2013), and another study has observed AHL-dependent induced SdiA-regulation of copper transport and type VI secretion system in this species (Sabag-Daigle et al., 2015). Furthermore, a study conducted with a *sdiA*-lacking *Cronobacter sakazakii* strain showed increased expression of capsule and lipopolysaccharide (LPS) synthesis genes (Cao et al., 2022).

The role of QS signalling in *K. pneumoniae* has only been the subject of a limited number of studies, and there is considerable inconsistency in the literature regarding AHL-regulated QS. For instance, some authors have proposed that *K. pneumoniae* is devoid of the *luxI* homologues for synthesis of AHLs (Subramoni and Venturi, 2009; Pacheco et al., 2021). However, other researchers have reported the production of AHLs in *K. pneumoniae* strains (Wang et al., 2006; Ngeow et al., 2013; Hosny and Fadel, 2021). With regard to SdiA, the only study found in the literature conducted by Pacheco et al. (2021) proposed that SdiA functions as a repressor of biofilm formation and fimbriae expression in *K. pneumoniae*. To conduct the study, the authors employed a *sdiA* transposon-based insertion mutant of the *K. pneumoniae* ATCC 10031 collection strain and examined the impact of *N*-octanoyl-L-homoserine lactone (C8-HSL) as an exogenous AHL on a microtiter-based biofilm formation cultivation model. In this study, the authors observed that the biofilm-repressing effect of the AHL was *sdiA*-dependent. However, a preliminary work carried out in our laboratory with several clinical strains about the effect of different AHLs on biofilm formation revealed no effect of C8-HSL and high variability on strain response (unpublished results). Therefore, a deeper understanding is needed in order to develop new anti-virulence strategies.

The aim of this study is to deeper our knowledge about the function of SdiA and AHL supplementation in virulence-related traits and biofilm formation of *K. pneumoniae*. The most active AHL was selected for evaluation of virulence phenotypes, including biofilm formation, capsular synthesis, serum resistance, and phage sensitivity, plus virulence assessment *in vivo* in *Galleria mellonella*. The *K. pneumoniae* strain selected for this study was KLEB-33, a multiresistant, hypermucoviscous and hyperbiofilm-forming clinical strain (Smith and Ahmer, 2003) that harbours several virulence genes that are characteristic of hypervirulent *K. pneumoniae* strains (Russo and Marr, 2019). The genetic and phenotypic characteristics of KLEB-33 render it an optimal model for the study of the emerging convergent *K. pneumoniae* strains, as the majority of the QS studies to date have been performed in collection strains. For comparative purposes, the aforementioned phenotypes were also studied in a non-virulent, low biofilm forming, and non-MDR ATCC 13883^T^ strain. The results of this study demonstrate that C6-HSL is the most effective AHLs on promoting biofilm formation. Additionally, C6-HSL and SdiA exert a dual influence on virulence phenotypes, operating both independently and hierarchically. The experiments conducted have facilitated a more profound comprehension of the QS mechanisms in *K. pneumoniae*.

## 2 Materials and methods

### 2.1 Bacterial strains and culture conditions

This study employed the *K. pneumoniae* ATCC 13883^T^ and KLEB-33 strains. KLEB-33 is a MDR hyper-biofilm-forming clinical strain harbouring hypervirulence genes used as convergent-model strain (Silva-Bea et al., 2024a). This strain was obtained from a previous study (Silva-Bea et al., 2024a) approved by the Institutional Ethics Committee (CEImPA 03/2018). The present study did not require to be reviewed or approved by an ethics committee. ATCC 13883^T^ is a low-biofilm forming non-MDR nor hypervirulent collection strain used for comparative purposes. The strains were routinely grown at 37 °C/200 rpm on 5 mL Lysogeny broth (LB) or LB agar (1.5% w/v). LB broth was supplemented with glucose 0.4% when required. Antibiotics were added when required, and synthetic AHL signals dissolved in acetonitrile at a concentration of 10 mg/mL, and comprising acyl chains with a carbon length of 4 to 18, including the oxo- substituted AHLs oxo-C4-HSL and oxo-C6-HSL, were added at a final concentration of 5, 2 or 0.2 µM, as required. An equal amount of solvent was added to the control cultures in all experiments involving AHL addition.

### 2.2 Construction of *sdiA* mutants

The *sdiA* gene was deleted from the ATCC 13883^T^ and KLEB-33 strains using the CRISPR/Cas9-based system with the pCas9KP-Apr and pSGKP-Km plasmids, as previously described (Wang et al., 2018). The sequences of the single-guide RNA (sgRNA) spacer, the single-stranded DNA (ssDNA) sequences employed for allelic knockout, and the primers used for mutation confirmation are shown in **Supplementary Table S1**. Completely removal of the gene was confirmed by Sanger sequencing.

Planktonic growth was assessed in 15 mL LB cultures, plus the effect of AHL addition. Briefly, cultures inoculated at an initial absorbance at 600 nm of 0.01 (Abs_600 nm_) were incubated at 200 rpm, 37 °C/24 h, with growth measured at 1, 2, 4, 6, 8, 10 and 24 h, in triplicate. The lineal relationship between Colony Forming Units (CFUs) and Abs_600 nm_ was confirmed using the Miles and Misra method (Miles et al., 1938), with the experiments being repeated twice.

### 2.3 Biofilm cultivation

*K. pneumoniae* KLEB-33 biofilms were cultivated in LB or LB+Glucose (0.4%) using the active attachment (AA) method as previously described (Silva-Bea et al., 2024b). Briefly, biofilms were grown for 24 h/37 °C in 12-well plates (VWR, 734-2778) in aerobiosis using a custom-made aluminium lid with glass coverslips (18x18 mm) attached as a substrate. Bacteria were inoculated at final Abs_600 nm_ of 0.05. The culture media and treatment were replaced at 12 h to facilitate the growth of adherent cells. The Rolling Biofilm Bioreactor (RBB) cultivation method (Romero et al., 2022) was also used to promote biofilm growth and maturation of KLEB-33 and ATCC 13883^T^ strains of *K. pneumoniae*. In this system bacteria were inoculated at a final Abs_600 nm_ of 0.01 and incubated at 37 °C/72 h, with media and treatment changes every 24 h. The biofilm biomass was quantified by staining with crystal violet (CV) (0.04%) and measuring the absorbance at Abs_590 nm_ after washing the coverslips with 33% acetic acid (Exterkate et al., 2010).

RBB biofilms were stained with Syto9 (ThermoFisher S34854) and subsequently imaged by confocal laser scanning microscopy (CLSM) (Leica Stellaris 8) to quantify biofilm height and coverage at 24, 48 and 72 h. Furthermore, 24 h biofilms were also examined for bacterial filamentation. To examine the composition and structure of biofilms, 48 h samples were also stained with YOYO™-1 iodide (ThermoFisher Y3601), Concanavalin A conjugated with Alexa Fluor^®^ 594 (ThermoFisher C11253) and lipophilic FM™ 4-64 (ThermoFisher, F34653) fluorescent dyes to stain biofilm extracellular DNA (eDNA), extracellular polysaccharides, and cell membranes, respectively. A total of six fields were collected per sample. Images were subsequently analysed using ImageJ (v1.54) and Leica Application Suite X Office (v1.4.6.28433).

### 2.4 Quorum quenching activity

Quorum quenching (QQ) activity was evaluated in accordance with the methodology previously described (Parga et al., 2023). Briefly, 500 µL of the filtrated (0.22 µm) supernatant and pellet (resuspended in PBS pH 6.5) portions of 15 mL 24 h LB culture samples were exposed to C6-HSL (10 µM) for 6, 12, 24 and 48 h. pH of samples was adjusted when necessary to 6.5 to prevent the spontaneous opening of the lactone ring. PBS pH 6.5 with C6-HSL (10 µM) was used as negative control. After incubation, 100 µL of each sample was added to wells prepared in soft LB agar plates (0.8%) with the biosensor *Chromobacterium subtsugae* CV026, and incubated at 30 °C/24 h. Pellet samples were filtrated to avoid contamination of biosensor. Absence of production of violacein by the biosensor was indicative of positive QQ activity. Biosensor was routinely grown in LB broth supplemented with kanamycin (25 µg/mL).

### 2.5 Percoll density gradient centrifugation and capsule staining

Percoll density gradient centrifugation was employed to quantify strain capsule expression, in accordance with the methodology described (Dorman et al., 2018). Bacteria were adjusted to Abs_590 nm_ = 1, collected from overnight cultures by centrifugation and resuspended in 2 mL PBS. The bacterial suspension was added to the top of a Percoll density gradient comprising 80, 60, 40 and 20% solutions in PBS to separate the bacterial fractions after centrifugation (2600 g) at 4 °C/20 min (9x acceleration; 1x deceleration). The distance between the bottom of the tube and the cell layer was measured. Capsule staining was also conducted using the Maneval method (Hughes and Smith, 2007).

### 2.6 Human serum sensitivity

The susceptibility to human serum was assessed as previously described (Dorman et al., 2018; Lv et al., 2022). Briefly, the bacterial inoculum was adjusted to an Abs_600 nm_ of 1 in PBS, and 100 µL of bacterial suspension was added to 200 µL of pre-warmed (37 °C) human serum (Merk, S7023). Mixture was incubated at 37 °C/2 h. Colony-forming units per millilitre (CFU/mL) were determined on LB agar plates.

### 2.7 Phage susceptibility

Phage susceptibility was evaluated using the specific lytic phage *Webervirus kpv33d1* (Sonia Rey et al., unpublished) as previously described (Xie et al., 2018). Briefly, the bacterial inoculum was grown in LB to an Abs_600 nm_ of 0.5, diluted 1/5, and 10 µL were added to each well containing 90 µL of pre-diluted phage, resulting in a final Abs_600 nm_ of 0.01 (approximately 10^6^ CFU/mL). Phage at 10^10^ Plate Forming Units (PFU)/mL was serially diluted 1/10 to up to 9 times in 90 µL LB in 96-well U-bottom plates, from a Multiplicity Of Infection (MOI) of 10^3^ to 10^-5^. A gas-permeable membrane (Breathe-Easy^®^, Z380059) was applied, and the plate was incubated at 37 °C/24 h, with Abs_600 nm_ readings of the cultures every 30 min.

### 2.8 *Galleria mellonella* infection model

The virulence was evaluated using the *G. mellonella* survival assay, as previously described (Insua et al., 2013; Gato et al., 2020). Briefly, 15 larvae (300 - 400 mg body weight) were injected with 10 μL of a suspension containing 10^3^, 10^5^, or 10^7^ CFUs in PBS. Larvae injected with an equal volume of sterile PBS or PBS plus C6-HSL (5 µM) were used as controls. Larvae were incubated at 37 °C in the dark and mortality was monitored every 24 h for up to 3 days.

### 2.9 Statistical analysis

Statistical analyses were conducted using GraphPad Prism 8.3.0. First, a Shapiro-Wilk test was used to ascertain whether the data exhibited a normal distribution. If normal, an analysis of variance (ANOVA) or a Student’s t-test was conducted. Alternatively, a Kruskal-Wallis or a Mann-Whitney test was performed, depending on whether there were more than two groups or only two groups, respectively. The significance values indicated by asterisks in the graphs presented in this paper are as follows: * = p<0.05; ** = p<0.005; *** = p<0.0005; and **** = p<0.00005.

## 3 Results and discussion

### 3.1 C6-HSL exhibited the greatest effect in promoting biofilm formation in AA cultivation system

Several reports indicate that SdiA can be activated by different AHLs in *E. coli* and *Salmonella* (Michael et al., 2001; Ahmer, 2004; Janssens et al., 2007; Panchal et al., 2024). It is therefore necessary to perform a screening of different AHLs in a robust and repeatable biofilm cultivation method to elucidate the function of SdiA in biofilm formation following AHL addition in *K. pneumoniae*. To this end, the effect of three short-chain homoserine lactones (C4-HSL, C6-HSL and C8-HSL), two oxo- substituted short-chain homoserine lactones (oxo-C4-HSL and oxo-C6-HSL) and five long-chain homoserine lactones (C10-HSL, C12-HSL, C14-HSL, C16-HSL and C18-HSL) on the AA biofilm cultivation system in the hyper-biofilm-forming strain KLEB-33 was investigated. The AA system has been already demonstrated to be a reliable and repeatable system for biofilm studies in this strain (Silva-Bea et al., 2024b).

**Figure 1:**
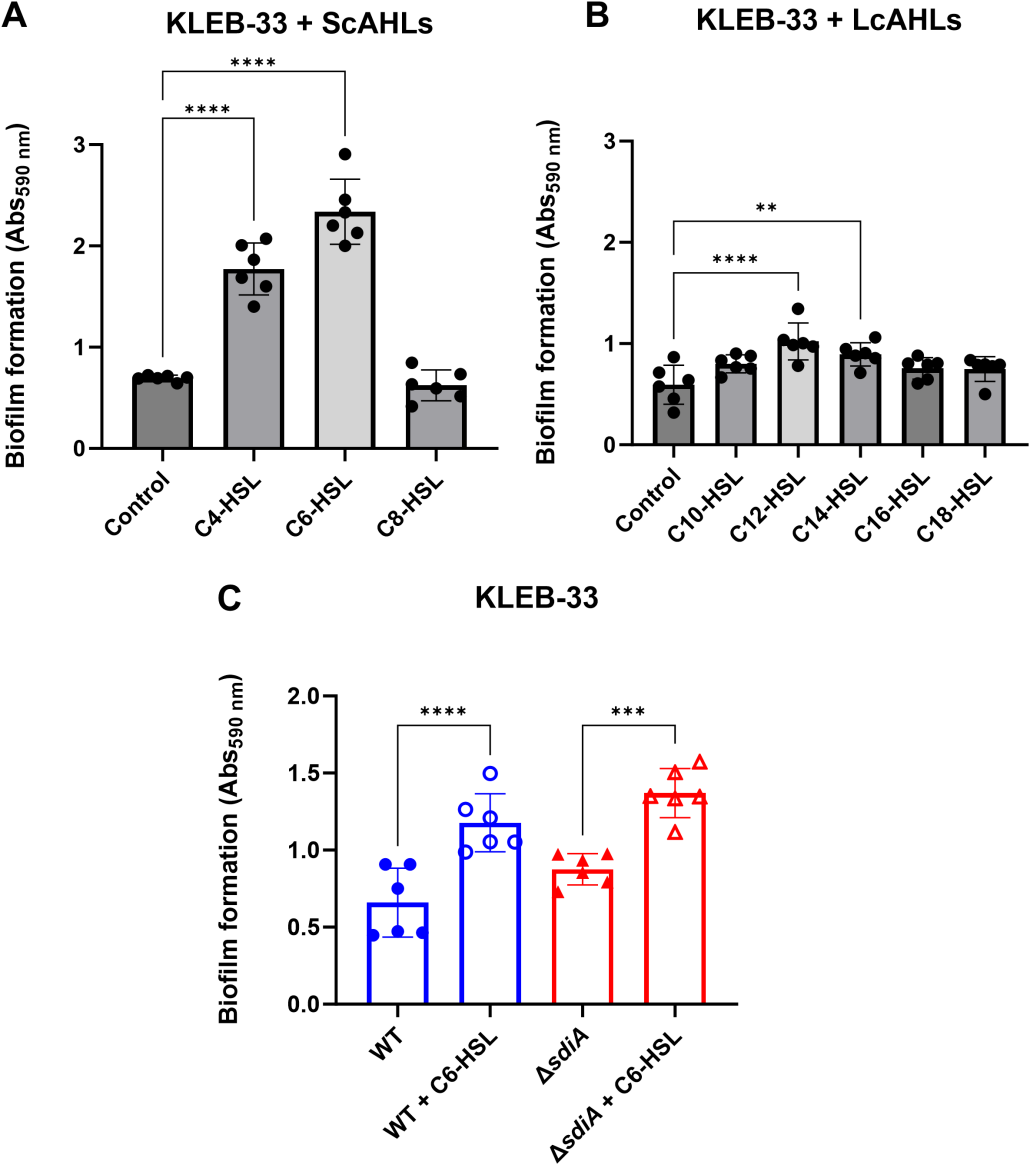
Effect of AHL addition (5 µM) on biofilm formation by *K*. *pneumoniae* KLEB-33 on glass coverslips in the Active Attachment (AA) biofilm model. The quantification of biofilm formation was conducted using CV staining, which was subsequently dissolved with 33% acetic acid and the absorbance measured at 590 nm (Abs_590 nm_). No effect was observed at 2 µM in previous experiments (**Supplementary Figure S1**). The impact of each AHL individually at 5 µM was examined for short-chain AHLs (ScAHLs) **(A)** and long-chain AHLs (LcAHLs) **(B)** in comparison to the solvent control. The repeatability of the C6-HSL effect was validated in subsequent experiments conducted on wild-type and *sdiA*-deficient KLEB-33 strains **(C)**. All the experiments were conducted in duplicate (N = 3).

The results showed that in the clinical strain KLEB-33 the AHLs C4-HSL, oxo-C4 and C6-HSL at 5 µM exhibited a substantial impact on biofilm formation in the absence of glucose (**Figure 1A**; **Supplementary Figure S1A**), with C12-HSL and C14-HSL also exhibiting a significant effect, albeit with a considerably lower magnitude than that observed for the short-chain AHLs (**Figure 1B**). In the presence of glucose 0.4%, oxo-C6-HSL also had a significant effect on this phenotype (**Supplementary Figure S1A**). However, none of the tested AHLs exhibited an effect on biofilm formation at 2 µM (**Supplementary Figure S1B**), although it should be noted that other phenotypes further examined in this study could respond to this lower AHL concentration. The influence of AHL provision on ATCC 13883^T^ strain was also assessed in the AA cultivation system (**Supplementary Figure S1C**). However, its inherent low biofilm-forming capacity hindered the detection of significant differences in this biofilm model system. Following these results, C6-HSL in LB without glucose was selected for the subsequent experiments, as it exhibited the most pronounced contrast in comparison with the control cultures.

In contrast with observations made by Pacheco et al. (2021), our findings did not indicate a strong promoting effect on biofilm formation of C8-HSL. This suggests a high variability on the response to AHLs among different strains. Given the significant biofilm-promoting impact of C6-HSL observed in our experiments, we sought to ascertain whether this effect could be attributed to its direct interaction with SdiA. To this end, a Δ*sdiA* strain was constructed in KLEB-33 and was cultivated under C6-HSL supplementation. A significant increase in biofilm formation was also observed in the *sdiA*-lacking strain in response to C6-HSL addition (**Figure 1C**), indicating that the biofilm-promoting effect of C6-HSL was independent of *sdiA*. Moreover, despite SdiA being described in the literature as a biofilm repressor (Pacheco et al., 2021; Mayer et al., 2023), in the AA biofilm cultivation system the experiments revealed only a slight, non-significant increase in biofilm formation on the Δ*sdiA* in comparison to its wild-type. Growth was monitored with/without AHL supplementation in shaken cultures to ascertain that *sdiA* deficiency and/or C6-HSL addition does not affect growth (**Supplementary Figure S2**).

It is noteworthy that the concentration of C6-HSL at which a significant impact on biofilm formation was observed is higher than the physiological AHL concentrations typically encountered in QS signalling species (Milton et al., 2001). Nevertheless, similar concentrations in the micromolar range have been used to elicit a biological effect in *K. pneumoniae* (Høyland-Kroghsbo et al., 2013; Gopu et al., 2016; Pacheco et al., 2021) and other bacterial species (Michael et al., 2001; Dyszel et al., 2010b) for the study of the role of SdiA. In the case of *K. pneumoniae*, the presence of AHL-degrading enzymes has previously been described (Chan, 2013), and the presence of QQ activity against C6-HSL was corroborated in the culture media of the strains used in this study (**Supplementary Table S2**), therefore, the high concentrations required to observe a phenotypic response could be also due to the partial inactivation of the AHLs. Nevertheless, and due to the high AHL concentration required to elicit a response, we cannot fully disregard the possibility that these molecules are acting through non-specific mechanisms, for example, by interacting with the cellular membranes, as reported previously for long-chain, oxo-substituted AHLs (Davis et al., 2010; Gahan et al., 2021).

### 3.2 The formation and maturation of *K. pneumoniae* biofilms are influenced by SdiA and C6-HSL in an opposing and hierarchically organised manner

**Figure 2:**
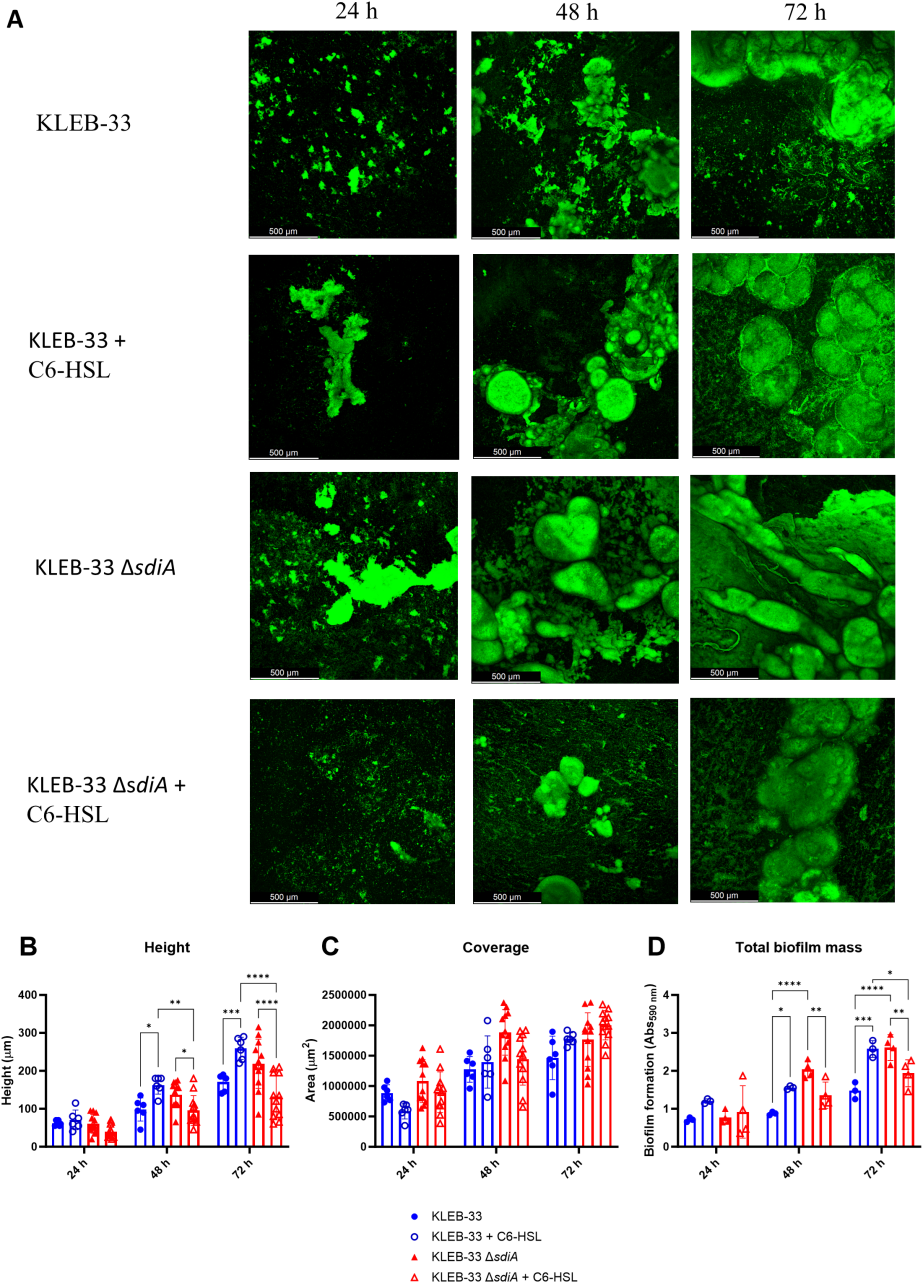
Impact of *sdiA* mutation and AHL addition (5 µM) on biofilm formation in *K*. *pneumoniae* KLEB-33. **(A)** Representative CLSM images (N = 3) of biofilms obtained after 24, 48 and 72 hours of incubation in the RBB cultivation system and staining with Syto9 fluorescent dye. **(B, C)** Quantification of height and biofilm coverage from confocal images using ImageJ (v1.54) image analysis software. **(D)** Crystal Violet quantification (N = 3) of biofilm biomass of wild-type and Δ*sdiA K. pneumoniae* KLEB-33 strains after treatment with C6-HSL (5 µM) or the solvent control (acetonitrile). The experiment was repeated twice.

In order to evaluate the impact of *sdiA* mutation and C6-HSL supplementation on biofilm structure and maturation, we also employed the rolling biofilm bioreactor (RBB) system (Romero et al., 2022). This system permits the cultivation of biofilms over extended periods and the generation of highly matured biofilms with high reproducibility, even in strains with low biofilm-forming capabilities, such as ATCC 13883^T^. Even though little differences were observed in the early stages of biofilm formation, the results obtained with the RBB system demonstrated that the KLEB-33 Δ*sdiA* strain exhibited a higher biofilm maturation in comparison to the wild-type strain. This was evidenced by the earlier formation of mushroom-like structures in the Δ*sdiA* strain after 48 hours of incubation and a higher biofilm biomass (**Figure 2**). Our observations in the RBB cultivation system are in accordance with a role of SdiA as a biofilm-repressor, as previously described in the literature (Sabag-Daigle et al., 2015; Pacheco et al., 2021; Mayer et al., 2023; Schwieters and Ahmer, 2024). Nevertheless, no notable differences were observed between the wild-type and Δ*sdiA* strains at 24 hours of incubation, as happened in the AA cultivation system, as the 24-hour biofilms had not yet reached a stage of development sufficient to manifest such differences. Indeed, comparable levels of biofilm formation were recorded in the AA and RBB cultivation systems for both strains following a 24-hour incubation period (**Figures 1**, **2**). On the contrary, no significant increase in biofilm formation was observed in the ATCC 13883^T^ Δ*sdiA* strain. This finding may again be attributed to the inherent lower biofilm formation ability of the strain.

The addition of C6-HSL (5 μM) also promoted the maturation of the biofilm in the KLEB-33 and ATCC 13883^T^ wild-type strains after 48 hours of incubation (**Figure 2**; **Supplementary Figure S3**). However, in the roller biofilm bioreactor no such promotion effect was observed when AHL was added to the Δ*sdiA* for both strains studied, and these observations were corroborated by the quantification of the total biofilm biomass and thickness (**Supplementary Figure S3**, **Figure 2**). These results show that in the wild-type strain, SdiA functions as a repressor of genes involved in biofilm maturation, as previously reported (Pacheco et al., 2021; Mayer et al., 2023). However, this effect is negated in the presence of AHL, resulting in a biofilm maturation-promoting effect only when *sdiA* is present. The observed phenotype is only evident in the absence of *sdiA*, suggesting a hierarchical regulatory relationship between SdiA and C6-HSL.

**Figure 3:**
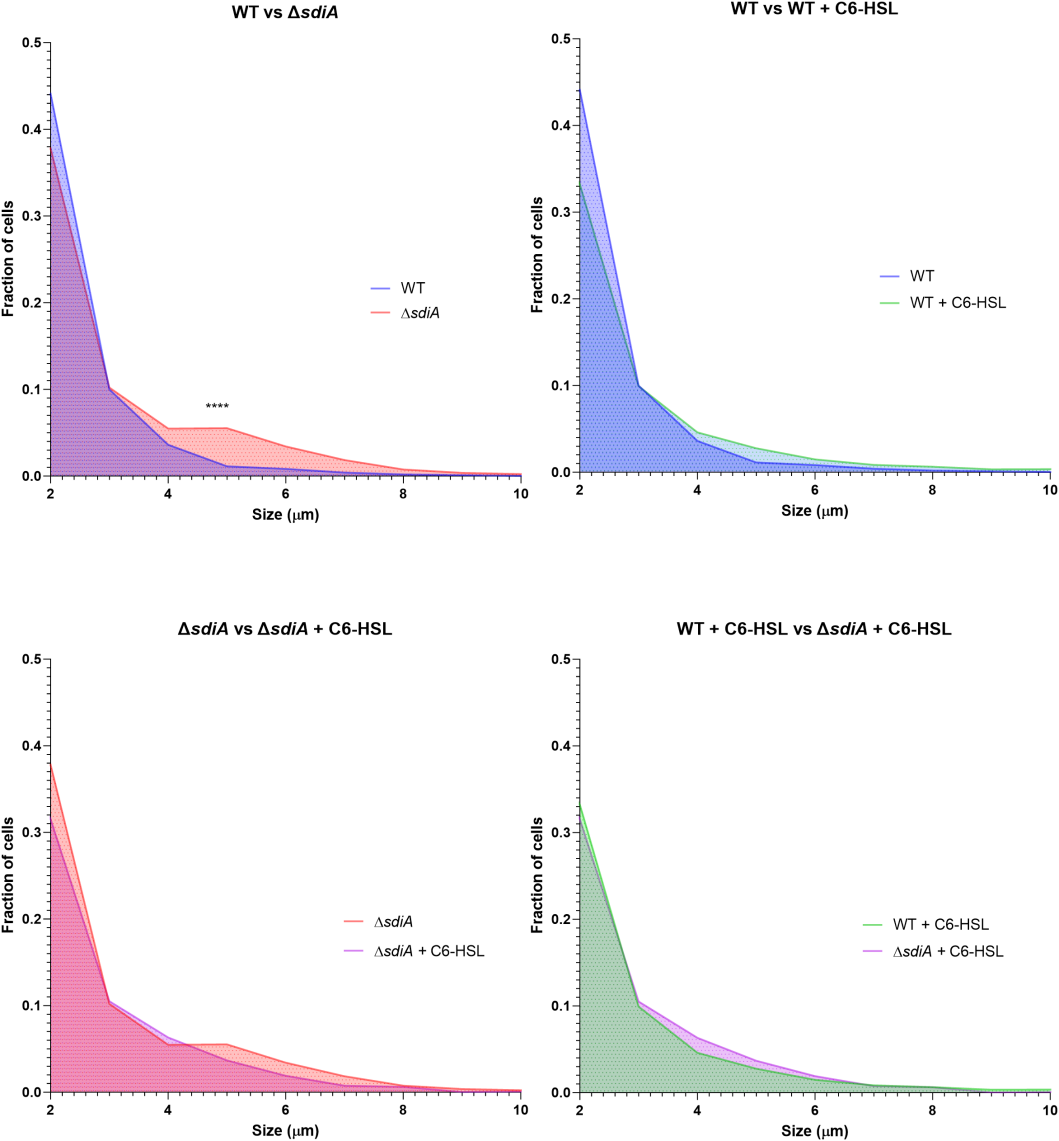
Distribution of cell size fractions in biofilms of *K. pneumoniae* KLEB-33 wild-type (WT) and Δ*sdiA* strains cultivated in the RBB system for 24 hours with supplementation of C6-HSL (5 µM) or the solvent control (acetonitrile). Cell sizes were measured using the ImageJ software (version 1.54). A total of 12 images were analysed per condition and sample (N = 3). The experiment was repeated twice.

Additionally, a significant increase in cell filamentation was observed in biofilms of the KLEB-33 Δ*sdiA*, a finding that aligns with previous observations (Pacheco et al., 2021) (**Figure 3**; **Supplementary Figure S4**). The formation of filamented bacteria has been documented in the literature as a mechanism that contributes to the persistence and colonisation of surfaces (Abell-King et al., 2022). Therefore, the elevated filamentation rates observed in the Δ*sdiA* strain are consistent with its enhanced capacity for biofilm formation (**Figure 2**). However, no differences were observed following C6-HSL supplementation, indicating that cell filamentation is dependent on SdiA, yet not triggered by AHL signalling. The AHL-independent effect of s*diA* has been reported before (Sabag-Daigle et al., 2015; Dyszel et al., 2010b; Schwieters and Ahmer, 2024).

**Figure 4:**
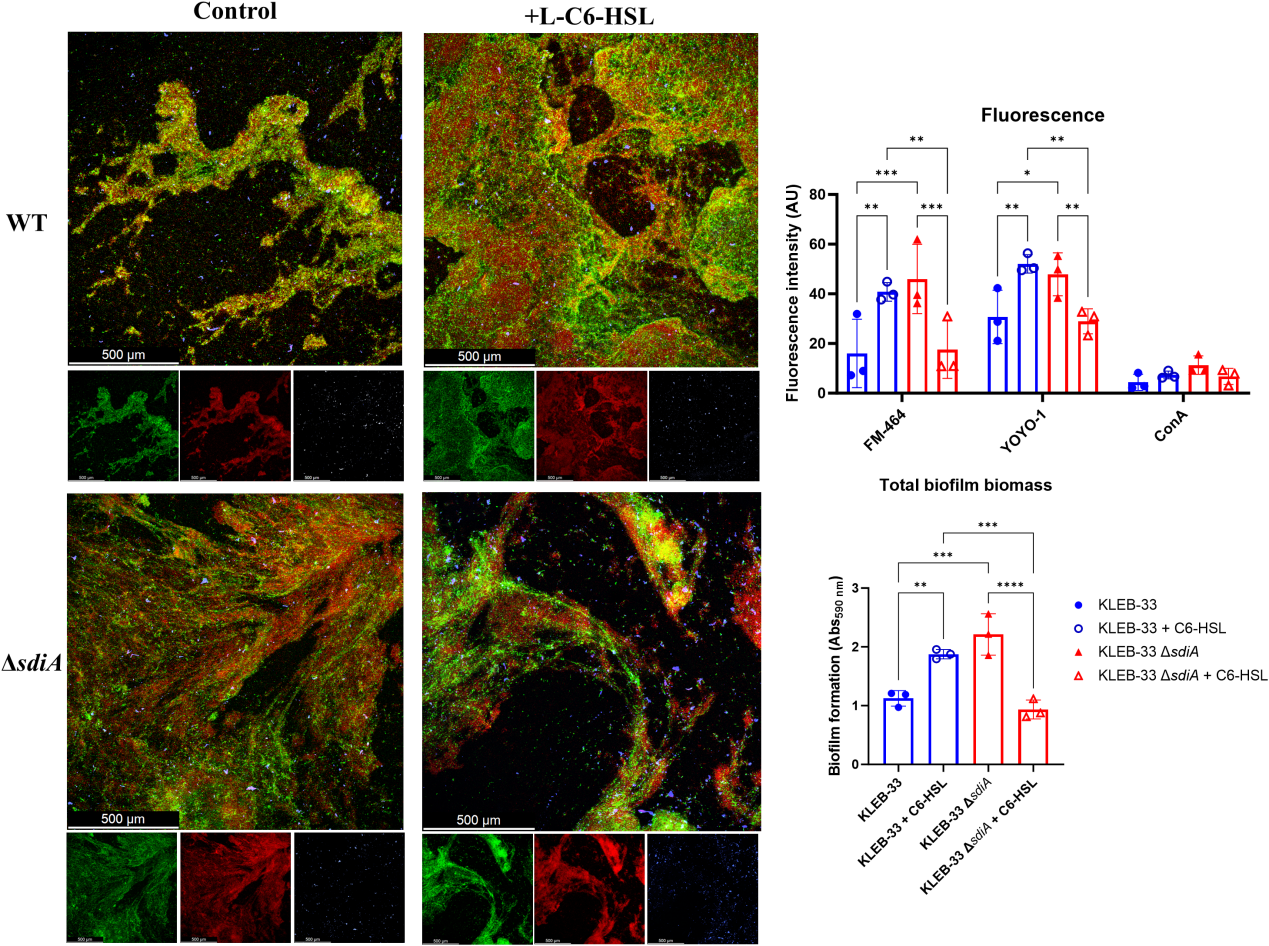
Representative CLSM (Leica Stellaris 8) images of RBB 48 hours biofilms of WT and Δ*sdiA K. pneumoniae* KLEB-33 treated with C6-HSL (5 µM) or the solvent control (acetonitrile). Biofilms were stained with YOYO-1 (eDNA, green), FM-464 (cell membranes, red), and Concanavalin A conjugated with AlexaFluor594 (exopolysaccharide, blue). A total of three fields were recorded in CLSM for each biofilm sample. Repeatability of the experiments was corroborated with total biomass quantification (N = 3) with CV staining (Abs_590nm_). Quantification of fluorescence intensity (AU: arbitrary units) was performed with ImageJ (v1.54).

In light of the observed alterations in biofilm maturation and structure following *sdiA* mutation and AHL supplementation, we sought to investigate whether *sdiA* deficiency or AHL signalling could influence the biofilm matrix composition in *K. pneumoniae*, as previously described for other pathogens (Das and Manefield, 2012; Yang and Lan, 2016). To this end, 48-hour RBB biofilms of KLEB-33 were fluorescently stained with YOYO-1 (eDNA), FM 4-64 (cell membranes) and Concanavalin A conjugated with AlexaFluor594 (ConA, extracellular polysaccharides). The results demonstrated a significant increase in fluorescence intensity for both the YOYO-1 and FM 4-64 signals (**Figure 4**) in the same conditions where enhanced biofilm formation was recorded (**Figure 2**: WT + C6-HSL and Δ*sdiA*). However, the ConA staining did not yield a strong fluorescence signal, which could suggest that eDNA may be the principal component of the biofilm matrix (**Supplementary Figure S5**). The function of eDNA as a crucial component of the biofilm matrix, facilitating the development of biofilms through the formation of adhesive “webs” (**Supplementary Figure S5**) that enhance cell cohesion and adhesion, has been previously postulated in a variety of bacteria (Mann et al., 2009; Campoccia et al., 2021; Romero et al., 2022). However, no clear relationship could be identified between SdiA or AHL addition and eDNA. This suggests that the regulation of this matrix component is not dependent on these regulatory systems. Moreover, and although other researchers have reported elevated polysaccharide production in a *sdiA*-deficient *C. sakazakii* strain (Cao et al., 2022), no changes in exopolysaccharide abundance were observed in CLSM biofilms images (**Figure 4**).

### 3.3 QS-induced changes in biofilm formation have no correlation with capsule production in *K. pneumoniae*

**Figure 5:**
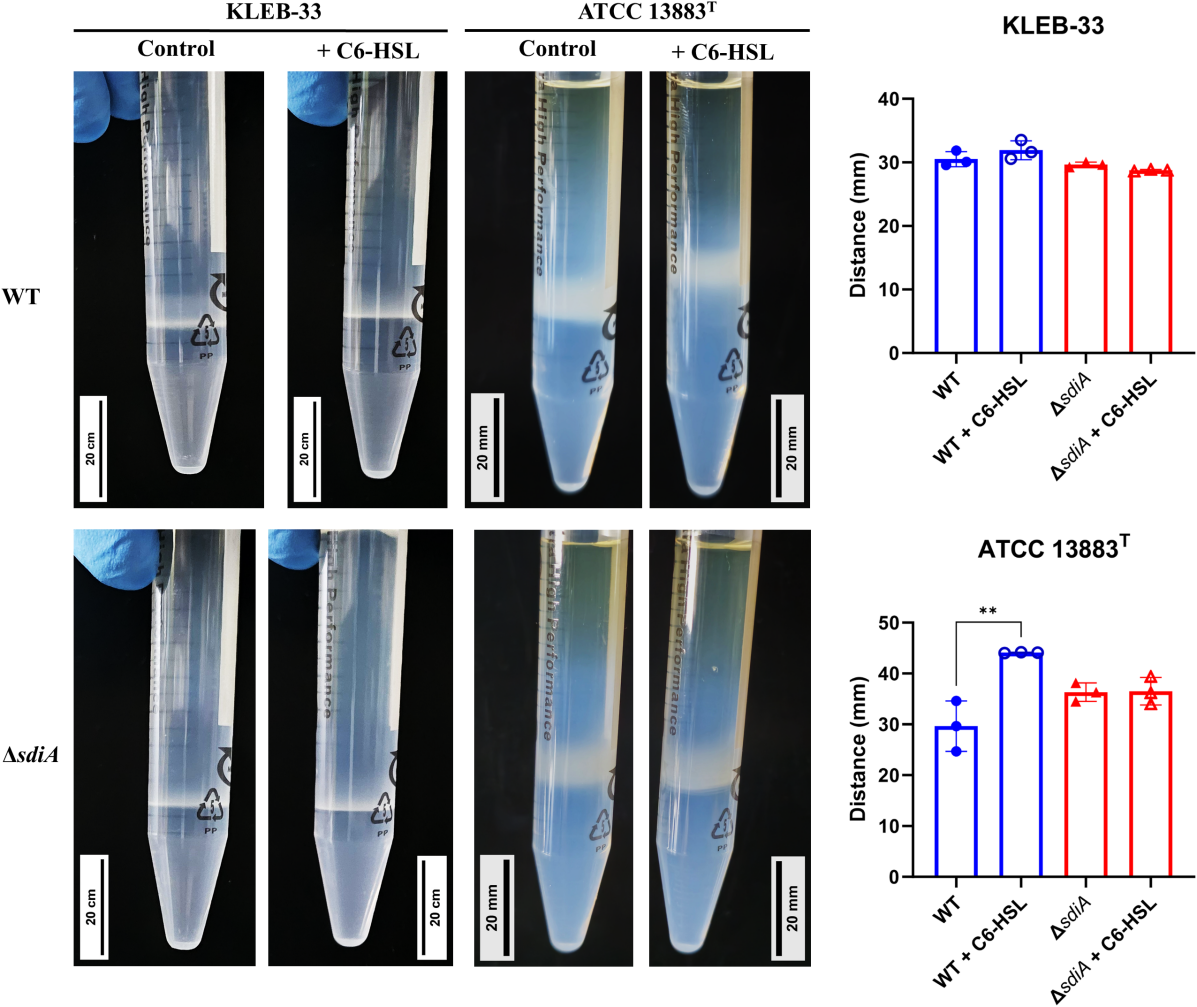
Percoll density gradient analysis conducted on *K*. *pneumoniae* KLEB-33 and ATCC 13883^T^ wild-type (WT) and *ΔsdiA* strains with and without the addition of AHL (5 µM). Representative images are presented in left side, while histograms of the obtained measurements for bacterial cell layer height (N = 3) are shown in the right side. Experiment was repeated twice. An equal amount of solvent was added to the control cultures.

To further examine the mechanism underlying the alterations in biofilm formation observed, we postulate that the supplementation of C6-HSL or the mutation of *sdiA* (which is associated with high biofilm formation) may be related to a reduction in capsule production. This is based on the findings of other researchers who have demonstrated that high-capsule-producing bacteria are more likely to be low biofilm formers, as the capsule polysaccharides have been shown to interfere with adhesion (Nunez et al., 2023). Consequently, a semi-quantitative analysis of the capsule production was conducted using Percoll density gradient centrifugation. This method enables the macroscopic differentiation of high and low capsule-producing bacteria based on their flotation characteristics (Dorman et al., 2018). Additionally, the capsules of the strains under study were subjected to staining using the Maneval’s method (Hughes and Smith, 2007). The results revealed differences between KLEB-33 and ATCC 13883^T^ strains, as a thicker band (**Figure 5**) and capsule (**Supplementary Figure S6**) was observed in the collection strain. However, no appreciable differences in capsule production between the wild-type and the Δ*sdiA* for both strains studied were observed (**Supplementary Figure S6**; **Figure 5**). Regarding the addition of C6-HSL, a significant increase in capsule production was recorded in the wild-type strain of ATCC 13883^T^, whereas no effect was observed in KLEB-33 floatability (**Figure 5**), which is likely attributable to its relatively lower capsule production compared to the ATCC 13883^T^ strain (**Supplementary Figure S6**, **Figure 5**). In fact, the low capsule production of KLEB-33 is consistent with its higher biofilm formation compared with ATCC 13883^T^ strain, a low biofilm-forming and high capsule-producing strain (Silva-Bea et al., 2024b). However, it seems that the C6-HSL induced capsule production does not affect biofilm formation, as we observed higher biofilm formation with AHL supplementation.

### 3.4 Serum complement killing in *K. pneumoniae* is inversely affected by SdiA and AHL supplementation

**Figure 6:**
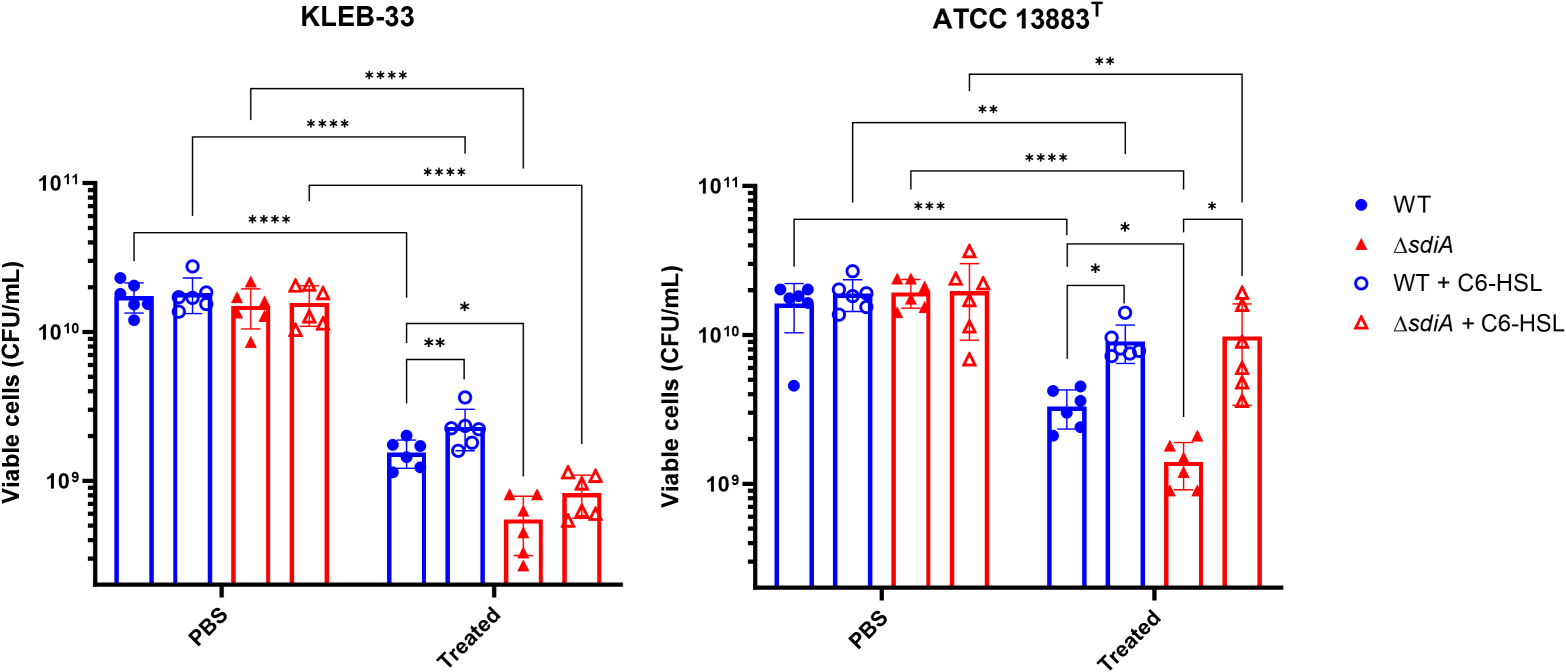
Serum resistance assays of *K*. *pneumoniae* KLEB-33 (left) and ATCC 13883^T^ (right) wild-type (WT) and Δ*sdiA* strains following exposure to human serum for 2 hours, plus the effect of C6-HSL supplementation (5 µM). The control samples represent non-serum-treated bacteria in PBS. The experiment was repeated twice with N = 3. An equal amount of solvent was added to the control cultures.

Although the Percoll method and Maneval’s staining did not reveal macroscopic differences in capsule production following *sdiA* mutation, we sought to identify a phenotype that could depend on capsule production and be sufficiently sensitive to detect differences in capsule biosynthesis. To this end, we conducted human serum survival assays as this method is employed by some authors as an indirect means of assessing capsule production (Lv et al., 2022). This is because capsule biosynthesis has been associated with serum and complement-killing resistance (González- et al., 2021). The results of our experiments showed that the *sdiA* knockout strains exhibited significantly elevated serum sensitivity in both strains under study (**Figure 6**), thereby indicating that SdiA plays a role in this process.

The reduced serum resistance exhibited by the Δ*sdiA* strains may be linked to modifications in capsule production, which could also account for the increased biofilm formation. In fact, KLEB-33 has shown higher serum sensitivity than ATCC 13883^T^ strain (**Figure 6**), showing a correlation with low capsule production and high biofilm formation, as described in the literature (Lv et al., 2022; Nunez et al., 2023). However, an alternative hypothesis is that an SdiA deficiency in *K. pneumoniae* may lead to alterations in other components of the cell surface that affect serum resistance. For instance, a number of studies indicates that LPS upregulation contributes to higher serum and phage sensitivity (González- et al., 2021; Majkowska-Skrobek et al., 2021; Tang et al., 2023). Moreover, the diminished serum resistance observed in the Δ*sdiA* strains is consistent with the findings of previous studies conducted in *Salmonella* spp (Lindsay and Ahmer, 2005). Additionally, type-1 fimbriae were found to be overexpressed in a *sdiA*-deficient strain of *K. pneumoniae* (Pacheco et al., 2021), and its overexpression has been linked to detrimental effects on complement survival in *E. coli* (Huja et al., 2014). Conversely, the addition of C6-HSL increased serum resistance independently of the presence of SdiA (**Figure 6**). Therefore, our experiments further support that C6-HSL supplementation promotes capsule production, a factor related with complement killing reduction (Dorman et al., 2018), as evidenced by the observation of enhanced serum resistance and a heightened band in Percoll experiments in the ATCC 13883^T^ strain.

### 3.5 SdiA deficiency increases phage sensitivity in *K. pneumoniae*

**Figure 7:**
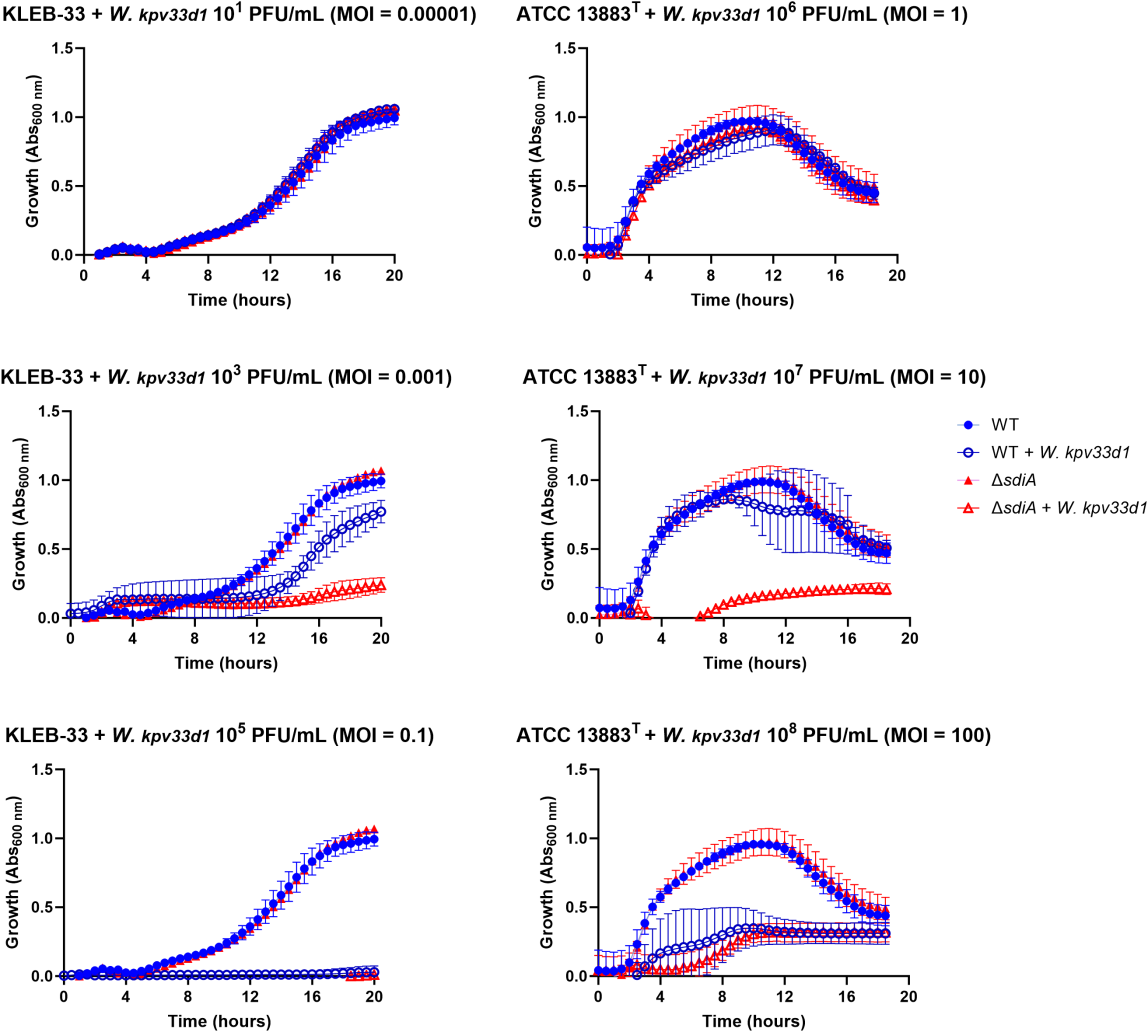
Susceptibility assays of *K. pneumoniae* KLEB-33 and ATCC 13883^T^ wild-type (WT) and Δ*sdiA* strains (10^6^ CFU/mL) to *Webervirus kpv33d1* phage. A dose-response assay was conducted using 96-well microtiter plates and varying MOI values. Experiments were performed twice for each strain separately (N = 3). No effect of C6-HSL addition (5 µM) was observed (**Supplementary Figure S7**).

The quantification of capsule production and complement-killing resistance experiments suggested that SdiA may affect cell surface components. Furthermore, a study conducted in *E. coli* demonstrated that SdiA plays a role in bacteriophage sensitivity through an AHL-dependent mechanism (Ghosh et al., 2009). Accordingly, an experiment was conducted to investigate whether modifications of the cell surface resulting from QS signalling could influence the susceptibility to phage infection. The infectivity of *Webervirus kpv33d1*, a high lytic wastewater-derived phage isolated against KLEB-33 strain (Sonia-Rey et al., unpublished), was tested on the wild type and genetically modified strains lacking the *sdiA* gene with or without AHL supplementation. Different levels of phage multiplicity of infection (MOI) were used for KLEB-33 (0.00001 to 0.1) and ATCC 13883^T^ (1 to 100) strains due to differences in phage specificity (**Figure 7**). The deletion of the *sdiA* gene resulted in higher phage susceptibility in comparison to the wild type strain in the presence of a phage MOI of 0.001 and 10 in the KLEB-33 and ATCC 13883^T^ strains, respectively. The addition of C6-HSL at concentrations of 5, 2 and 0.2 µM did not result in any observable effect on phage susceptibility in KLEB-33 (**Supplementary Figure S7**). These findings are consistent with those previously observed in *E. coli* (Ghosh et al., 2009) and also align with the results of serum resistance experiments, as evidenced by the heightened sensitivity of the Δ*sdiA* strains compared to the parental strains. The observation of higher serum and phage sensitivity of Δ*sdiA* may be also related to the higher filamentation rates observed (**Figure 3**), with higher cell surface being exposed to complement killing and phage attachment. However, the fact that C6-HSL addition has no effect on phage infection provides further support for a regulatory role of SdiA independent of C6-HSL.

### 3.6 C6-HSL signalling increases *Galleria mellonella* survival after infection with *sdiA*-deficient *K. pneumoniae*

**Figure 8:**
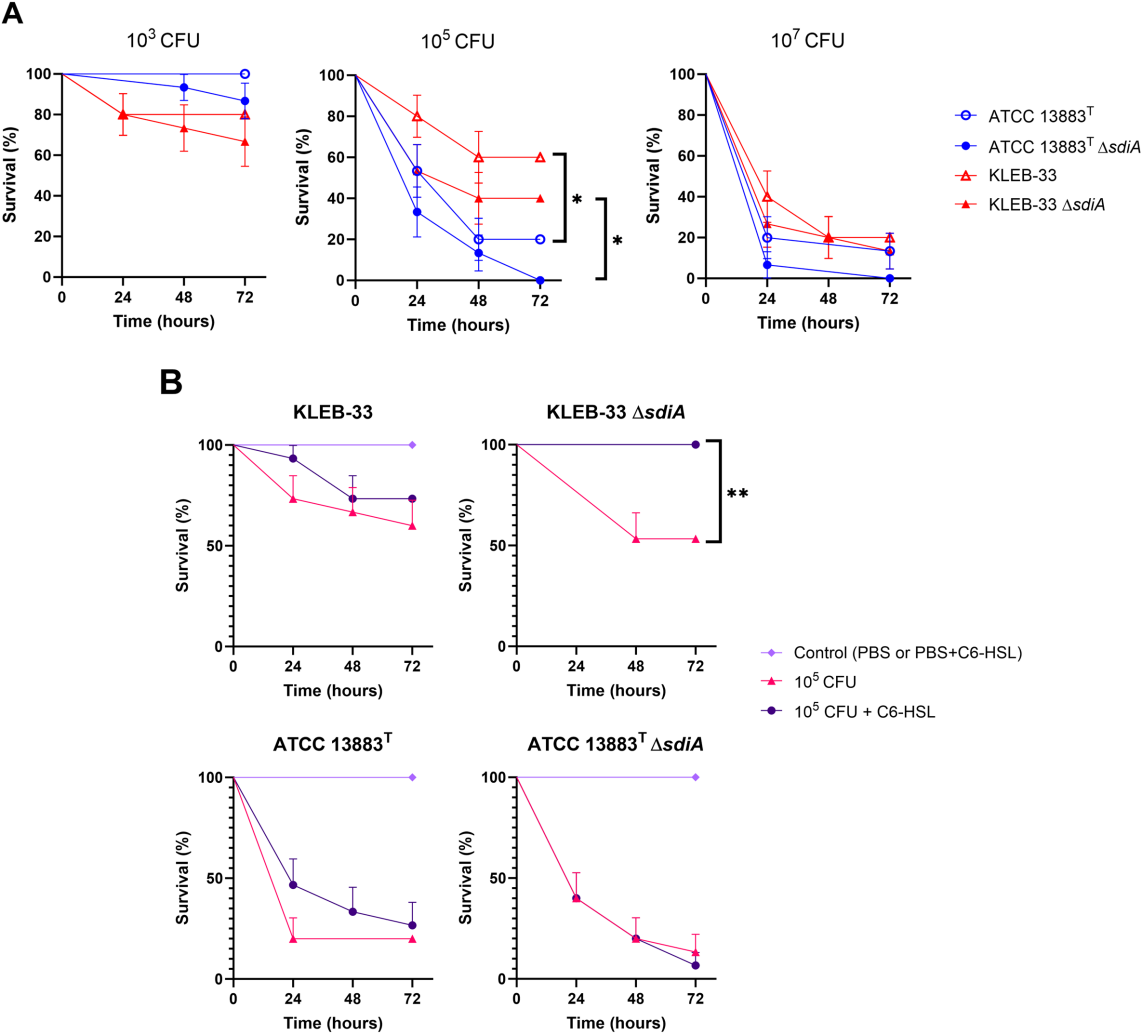
Survival analysis of *Galleria mellonella* following infection with *K. pneumoniae* KLEB-33 and ATCC 13883^T^ wild-type and Δ*sdiA* strains. The survival of the insects was recorded at 24-hour intervals up to 72 hours. The experiments were repeated twice. A total of 5 larvae per condition and sample (N = 3) was used. The results of the comparison between the concentrations of the strains **(A)** and the effect of C6-HSL addition (5 µM) **(B)** are presented. An equal amount of solvent was added to the control cultures.

In order to test how the mutation of *sdiA* or C6-HSL supplementation could affect virulence *in vivo*, we examined whether strains lacking SdiA could exhibit higher mortality rates in the *G. mellonella* infection model. As a result, despite the mortality rates recorded daily being consistently higher in the mutant, no statistically significant differences were observed between the wild-type and *ΔsdiA* strains for both KLEB-33 and ATCC 13883^T^ (**Figure 8A**). Regarding the addition of C6-HSL, a markedly reduced virulence was observed in the KLEB-33 *ΔsdiA* strain at a dose of 10^5^ CFU in the presence of AHL in comparison with the absence of AHL supplementation (**Figure 8B**). However, C6-HSL had no effect on virulence in either of the wild-type strains. Again, these results seem to indicate a separate regulatory pathway for SdiA and AHLs. The effect of decreased virulence observed following AHL treatment of the *sdiA* mutant strain was not replicated in ATCC 13883^T^. This may be attributed to the higher mortality rates observed in *G. mellonella* infected with this strain in comparison with KLEB-33, which demonstrated significantly lower mortality rates despite being a hyper biofilm-forming strain harbouring additional virulence genes (Silva-Bea et al., 2024a). However, the *G. mellonella* model proved inadequate for differentiating between classic and hypervirulent *K. pneumoniae* strains, and thus may not be a reliable indicator of virulence in murine models or in humans in certain cases (Russo and MacDonald, 2020). Additionally, a recent report showed unexpectedly lower virulence in convergent *K. pneumoniae* strains (Kochan et al., 2023). The notable reduction in virulence observed in KLEB-33 *ΔsdiA* supplemented with C6-HSL is consistent with the lower biofilm formation observed in RBB biofilms. The biofilm formation levels of *ΔsdiA* supplemented with C6-HSL observed in RBB experiments are very similar to WT levels (**Figure 2**). However, the WT strain did not show low virulence in *G. mellonella* model. A study performed in *Salmonella* has already reported depletion of adherence ability to HeLa cells in the presence of C6-HSL only in the Δ*sdiA* (Askoura et al., 2021). Therefore, we speculate that C6-HSL may control the expression of virulence and biofilm-formation genes independently and in opposition to SdiA, following a hierarchical regulation. This would explain why the biofilm and virulence repressive effect of C6-HSL could only be observed when SdiA was absent. However, though potentially statistically significant with a higher number of animals, there is no data to suggest that the *in vivo* survival phenotypes are linked to any of the *in vitro* virulence assays tested.

## 4 Conclusions

The aim of this study was to elucidate the function of SdiA in the virulence and biofilm formation of *K. pneumoniae*, which is postulated to be the receptor of AHLs. To this end, we firstly observed that C6-HSL is the most active AHL among 10 different signals. Following, a series of phenotypes were characterised in *sdiA*-lacking strains and after the addition of the C6-HSL, which was identified as a biofilm-promoting factor in this bacterium. Our findings appear to indicate that SdiA and C6-HSL influence several traits, with some exhibiting a joint effect, but the majority displaying independent regulation.

The results confirm that SdiA plays a role in repressing biofilm formation, and that its absence was linked to a reduction in resistance to human serum and phage infection, as well as a notable promotion of cell filamentation. However, no impact was observed on macroscopic capsule synthesis. Conversely, the exogenous addition of C6-HSL was found to promote capsule production in a *sdiA*-dependent manner in one of the strains studied. Moreover, C6-HSL was observed to enhance serum resistance independently of SdiA. Nevertheless, no impact on phage sensitivity was noted. Regarding biofilm formation, C6-HSL has a promoting effect when SdiA is present, but is decreased in its absence. This observation is consistent with the virulence data recorded in *G. mellonella*, which is reduced following the addition of C6-HSL in the absence of SdiA. Additionally, neither SdiA nor C6-HSL affects the composition of the biofilm matrix.

In view of these findings, it seems reasonable to conclude that C6-HSL is not the primary ligand of SdiA and that they act independently in the *K. pneumoniae* strains under consideration. Our results indicate that SdiA and C6-HSL are involved in different pathways in some cases, and their effects may even be counteracted by hierarchical regulation. This is corroborated by the observation that certain effects of C6-HSL could only be observed in the absence of SdiA. It is important to note that some of the results were dependent on the strain of *K. pneumoniae* studied, as not all the phenotypes observed were consistent in both strains. Our study provides new insights in QS regulation in this pathogen, even so more experiments are necessary to continue with the characterisation of their respective ways of action.

## Data Availability

The raw data supporting the conclusions of this article will be made available by the authors, without undue reservation.

## References

[B1] Abell-King C. Costas A. Duggin I. G. Söderström B. (2022). Bacterial filamentation during urinary tract infections. PLoS Pathog. 18, e1010950. doi: 10.1371/journal.ppat.1010950 36454736 PMC9714745

[B2] Ahmer B. M. M. (2004). Cell-to-cell signalling in Escherichia coli and Salmonella enterica. Mol. Microbiol. 52, 933–945. doi: 10.1111/j.1365-2958.2004.04054.x 15130116

[B3] Ahmer B. M. M. Van Reeuwijk J. Timmers C. D. Valentine P. J. Heffron F. (1998). *Salmonella typhimurium* encodes an sdiA homolog, a putative quorum sensor of the LuxR family, that regulates genes on the virulence plasmid. J. Bacteriol. 180, 1185–1193. doi: 10.1128/JB.180.5.1185-1193.1998 9495757 PMC107006

[B4] Askoura M. Almalki A. J. Lila A. S. A. Almansour K. Alshammari F. Khafagy E. S.. (2021). Alteration of *Salmonella enterica* Virulence and Host Pathogenesis through Targeting sdiA by Using the CRISPR-Cas9 System. Microorganisms 9, 2564. doi: 10.3390/microorganisms9122564 34946165 PMC8707642

[B5] Campoccia D. Montanaro L. Arciola C. R. (2021). Extracellular DNA (eDNA). A major ubiquitous element of the bacterial biofilm architecture. Int. J. Mol. Sci. 22, 9100. doi: 10.3390/ijms22169100 34445806 PMC8396552

[B6] Cao Y. Li L. Zhang Y. Liu F. Xiao X. Li X.. (2022). Evaluation of *Cronobacter sakazakii* biofilm formation after sdiA knockout in different osmotic pressure conditions. Food Res. Int. 151, 110886. doi: 10.1016/j.foodres.2021.110886 34980413

[B7] Chan K. G. (2013). Expression of *Klebsiella* sp. lactonase *ahlK* gene is growth-phase, cell-population density and *N* -acylhomoserine lactone independent. Front. Life Sci. 7, 132–139. doi: 10.1080/21553769.2013.833141

[B8] Das T. Manefield M. (2012). Pyocyanin promotes extracellular DNA release in pseudomonas aeruginosa. PLoS One 7, e46718. doi: 10.1371/journal.pone.0046718 23056420 PMC3466280

[B9] Davis B. M. Jensen R. Williams P. O’Shea P. (2010). The interaction of N-acylhomoserine lactone quorum sensing signaling molecules with biological membranes: implications for inter-kingdom signaling. PLoS One 5, e13522. doi: 10.1371/journal.pone.0013522 20975958 PMC2958149

[B10] Dorman M. J. Feltwell T. Goulding D. A. Parkhill J. Short F. L. (2018). The Capsule Regulatory Network of *Klebsiella pneumoniae* Defined by density-TraDISort. mBio 9, e01863–e01818. doi: 10.1128/mBio.01863-18 30459193 PMC6247091

[B11] Dyszel J. L. Smith J. N. Lucas D. E. Soares J. A. Swearingen M. C. Vross M. A.. (2010a). *Salmonella enterica* Serovar Typhimurium Can Detect Acyl Homoserine Lactone Production by *Yersinia enterocolitica* in Mice. J. Bacteriol. 192, 29–37. doi: 10.1128/JB.01139-09 19820103 PMC2798265

[B12] Dyszel J. L. Soares J. A. Swearingen M. C. Lindsay A. Smith J. N. Ahmer B. M. M. (2010b). E. coli K-12 and EHEC Genes Regulated by sdiA. PLoS One 5, e8946. doi: 10.1371/journal.pone.0008946 20126629 PMC2812512

[B13] Exterkate R. A. M. Crielaard W. Ten Cate J. M. (2010). Different response to amine fluoride by *Streptococcus mutans* and polymicrobial biofilms in a novel high-throughput active attachment model. Caries Res. 44, 372–379. doi: 10.1159/000316541 20668379

[B14] Gahan C. G. Patel S. J. Chen L. M. Manson D. E. Ehmer Z. J. Blackwell H. E.. (2021). Bacterial quorum sensing signals promote large-scale remodeling of lipid membranes. Langmuir 37, 9120–9136. doi: 10.1021/acs.langmuir.1c01204 34283628 PMC8450678

[B15] Gato E. Vázquez-Ucha J. C. Rumbo-Feal S. Álvarez-Fraga L. Vallejo J. A. Martínez-Guitián M.. (2020). Kpi, a chaperone-usher pili system associated with the worldwide-disseminated high-risk clone *Klebsiella pneumoniae* ST-15. Proc. Natl. Acad. Sci. 117, 17249–17259. doi: 10.1073/pnas.1921393117 32641516 PMC7382220

[B16] Ghosh D. Roy K. Williamson K. E. Srinivasiah S. Wommack K. E. Radosevich M. (2009). Acyl-homoserine lactones can induce virus production in lysogenic bacteria: an alternative paradigm for prophage induction. Appl. Environ. Microbiol. 75, 7142–7152. doi: 10.1128/AEM.00950-09 19783745 PMC2786502

[B17] González--Ferrer Peñaloza H. F. Budnick J. A. Bain W. G. Nordstrom H. R. Lee J. S.. (2021). Finding order in the chaos: outstanding questions in *Klebsiella pneumoniae* pathogenesis. Infect. Immun. 89, e00693–e00620. doi: 10.1128/IAI.00693-20 33558323 PMC8090965

[B18] Gopu V. Meena C. K. Murali A. Shetty P. H. (2016). Petunidin as a competitive inhibitor of acylated homoserine lactones in Klebsiella pneumoniae. RSC Adv. 6, 2592–2601. doi: 10.1039/C5RA20677D

[B19] Habyarimana F. Sabag-Daigle A. Ahmer B. M. M. (2014). The sdiA-regulated gene *srgE* encodes a type III secreted effector. J. Bacteriol. 196, 2301–2312. doi: 10.1128/JB.01602-14 24727228 PMC4054179

[B20] Hosny R. A. Fadel M. A. (2021). Detection of quorum sensing N-acyl-homoserine lactone molecules produced by different resistant *Klebsiella pneumoniae* isolates recovered from poultry and different environmental niches. Appl. Biochem. Biotechnol. 193, 3351–3370. doi: 10.1007/s12010-021-03605-w 34196919

[B21] Høyland-Kroghsbo N. M. Mærkedahl R. B. Svenningsen S. L. A. (2013). Quorum-sensing-induced bacteriophage defense mechanism. mBio 4, e00362–e00312. doi: 10.1128/mBio.00362-12 PMC3624510 23422409

[B22] Hughes R. B. Smith A. C. (2007). Capsule-Stain-Protocols (American Society for Microbiology). Available at: https://asm.org/ASM/media/Protocol-Images/Capsule-Stain-Protocols.pdf?ext=.pdf (Accessed February, 2024).

[B23] Huja S. Oren Y. Biran D. Meyer S. Dobrindt U. Bernhard J.. (2014). Fur is the master regulator of the extraintestinal pathogenic *Escherichia coli* response to serum. mBio 5, e01460–e01414. doi: 10.1128/mBio.01460-14 25118243 PMC4145685

[B24] Insua J. L. Llobet E. Moranta D. Pérez-Gutiérrez C. Tomás A. Garmendia J.. (2013). Modeling *Klebsiella pneumoniae* Pathogenesis by Infection of the Wax Moth *Galleria mellonella* . Infect. Immun. 81, 3552–3565. doi: 10.1128/IAI.00391-13 23836821 PMC3811777

[B25] Janssens J. C. A. Metzger K. Daniels R. Ptacek D. Verhoeven T. Habel L. W.. (2007). Synthesis of *N* -Acyl homoserine lactone analogues reveals strong activators of sdiA, the *Salmonella enterica* serovar typhimurium LuxR homologue. Appl. Environ. Microbiol. 73, 535–544. doi: 10.1128/AEM.01451-06 17085703 PMC1796990

[B26] Kochan T. J. Nozick S. H. Valdes A. Mitra S. D. Cheung B. H. Lebrun-Corbin M.. (2023). *Klebsiella pneumoniae* clinical isolates with features of both multidrug-resistance and hypervirulence have unexpectedly low virulence. Nat. Commun. 14, 7962. doi: 10.1038/s41467-023-43802-1 38042959 PMC10693551

[B27] Król J. E. Hall D. C. Balashov S. Pastor S. Sibert J. McCaffrey J.. (2019). Genome rearrangements induce biofilm formation in *Escherichia coli* C – an old model organism with a new application in biofilm research. BMC Genomics 20, 767. doi: 10.1186/s12864-019-6165-4 31640553 PMC6805351

[B28] Lee J. Maeda T. Hong S. H. Wood T. K. (2009). Reconfiguring the Quorum-Sensing Regulator SdiA of *Escherichia coli* To Control Biofilm Formation via Indole and *N* -Acylhomoserine Lactones. Appl. Environ. Microbiol. 75, 1703–1716. doi: 10.1128/AEM.02081-08 19168658 PMC2655446

[B29] Lindsay A. Ahmer B. M. M. (2005). Effect of *sdiA* on biosensors of *N* -acylhomoserine lactones. J. Bacteriol. 187, 5054–5058. doi: 10.1128/JB.187.14.5054-5058.2005 15995228 PMC1169494

[B30] Lv J. Zhu J. Wang T. Xie X. Wang T. Zhu Z.. (2022). The role of the two-component QseBC signaling system in biofilm formation and virulence of hypervirulent *Klebsiella pneumoniae* ATCC43816. Front. Microbiol. 13, 817494. doi: 10.3389/fmicb.2022.817494 35464966 PMC9019566

[B31] Majkowska-Skrobek G. Markwitz P. Sosnowska E. Lood C. Lavigne R. Drulis-Kawa Z. (2021). The evolutionary trade-offs in phage-resistant *Klebsiella pneumoniae* entail cross-phage sensitization and loss of multidrug resistance. Environ. Microbiol. 23, 7723–7740. doi: 10.1111/1462-2920.15476 33754440

[B32] Mambu J. Virlogeux-Payant I. Holbert S. Grépinet O. Velge P. Wiedemann A. (2017). An updated view on the Rck invasin of *Salmonella*: still much to discover. Front. Cell Infect. Microbiol. 7, 500. doi: 10.3389/fcimb.2017.00500 29276700 PMC5727353

[B33] Mann E. E. Rice K. C. Boles B. R. Endres J. L. Ranjit D. Chandramohan L.. (2009). Modulation of eDNA Release and Degradation Affects *Staphylococcus aureus* Biofilm Maturation. PLoS One 4, e5822. doi: 10.1371/journal.pone.0005822 19513119 PMC2688759

[B34] Mayer C. Borges A. Flament-Simon S. C. Simões M. (2023). Quorum sensing architecture network in Escherichia coli virulence and pathogenesis. FEMS Microbiol. Rev. 47, fuad031. doi: 10.1093/femsre/fuad031 37312272

[B35] Michael B. Smith J. N. Swift S. Heffron F. Ahmer B. M. M. (2001). SdiA of *Salmonella enterica* is a LuxR homolog that detects mixed microbial communities. J. Bacteriol. 183, 5733–5742. doi: 10.1128/JB.183.19.5733-5742.2001 11544237 PMC95466

[B36] Miles A. A. Misra S. S. Irwin J. O. (1938). The estimation of the bactericidal power of the blood. Epidemiol. Infect. 38, 732–749. doi: 10.1017/S002217240001158X PMC2199673 20475467

[B37] Milton D. L. Chalker V. J. Kirke D. Hardman A. Cámara M. Williams P. (2001). The luxM homologue VanM from *Vibrio Anguillarum* directs the synthesis of *N* -(3-hydroxyhexanoyl)homoserine lactone and *N* -hexanoylhomoserine lactone. J. Bacteriol. 183, 3537–3547. doi: 10.1128/JB.183.12.3537-3547.2001 11371516 PMC95229

[B38] Ngeow Y. Cheng H. Chen J. Yin W. F. Chan K. G. (2013). Short chain N-acylhomoserine lactone production by clinical multidrug resistant *Klebsiella pneumoniae* strain CSG20. Sensors 13, 15242–15251. doi: 10.3390/s131115242 24284772 PMC3871072

[B39] Nunez C. Kostoulias X. Peleg A. Y. Short F. Qu Y. (2023). A comprehensive comparison of biofilm formation and capsule production for bacterial survival on hospital surfaces. Biofilm 5, 100105. doi: 10.1016/j.bioflm.2023.100105 36711324 PMC9880390

[B40] Pacheco T. Gomes A. É. I. Siqueira N. M. G. Assoni L. Darrieux M. Venter H.. (2021). SdiA, a quorum-sensing regulator, suppresses fimbriae expression, biofilm formation, and quorum-sensing signaling molecules production in Klebsiella pneumoniae. Front. Microbiol. 12, 597735. doi: 10.3389/fmicb.2021.597735 34234747 PMC8255378

[B41] Panchal J. Prajapati J. Dabhi M. Patel A. Patel S. Rawal R.. (2024). Comprehensive computational investigation for ligand recognition and binding dynamics of sdiA: a degenerate LuxR -type receptor in Klebsiella pneumoniae. Mol. Divers. 28, 3897–3918. doi: 10.1007/s11030-023-10785-6 38212453

[B42] Papenfort K. Bassler B. L. (2016). Quorum sensing signal–response systems in Gram-negative bacteria. Nat. Rev. Microbiol. 14, 576–588. doi: 10.1038/nrmicro.2016.89 27510864 PMC5056591

[B43] Parga A. Muras A. Otero-Casal P. Arredondo A. Soler-Ollé A. Àlvarez G.. (2023). The quorum quenching enzyme Aii20J modifies *in vitro* periodontal biofilm formation. Front. Cell Infect. Microbiol. 13, 1118630. doi: 10.3389/fcimb.2023.1118630 36816581 PMC9932050

[B44] Rahmati S. Yang S. Davidson A. L. Zechiedrich E. L. (2002). Control of the AcrAB multidrug efflux pump by quorum-sensing regulator sdiA. Mol. Microbiol. 43, 677–685. doi: 10.1046/j.1365-2958.2002.02773.x 11929524

[B45] Romero M. Mayer C. Heeb S. Wattanavaekin K. Cámara M. Otero A.. (2022). Mushroom-shaped structures formed in *Acinetobacter baumannii* biofilms grown in a roller bioreactor are associated with quorum sensing–dependent Csu-pilus assembly. Environ. Microbiol. 24, 4329–4339. doi: 10.1111/1462-2920.15985 35352448 PMC9790458

[B46] Russo T. A. MacDonald U. (2020). The *Galleria mellonella* Infection Model Does Not Accurately Differentiate between Hypervirulent and Classical *Klebsiella pneumoniae* . mSphere 5, e00850–e00819. doi: 10.1128/mSphere.00850-19 31915230 PMC6952204

[B47] Russo T. A. Marr C. M. (2019). Hypervirulent *Klebsiella pneumoniae* . Clin. Microbiol. Rev. 32. doi: 10.1128/CMR.00001-19 PMC6589860 31092506

[B48] Sabag-Daigle A. Ahmer B. M. M. (2012). ExpI and PhzI are descendants of the long lost cognate signal synthase for sdiA. PLoS One 7, e47720. doi: 10.1371/journal.pone.0047720 23082201 PMC3474713

[B49] Sabag-Daigle A. Dyszel J. L. Gonzalez J. F. Ali M. M. Ahmer B. M. M. (2015). Identification of sdiA-regulated genes in a mouse commensal strain of Enterobacter cloacae. Front. Cell Infect. Microbiol. 5. doi: 10.3389/fcimb.2015.00047 PMC4444967 26075189

[B50] Sabag-Daigle A. Soares J. A. Smith J. N. Elmasry M. E. Ahmer B. M. M. (2012). The Acyl Homoserine Lactone Receptor, sdiA, of *Escherichia coli* and *Salmonella enterica* Serovar Typhimurium Does Not Respond to Indole. Appl. Environ. Microbiol. 78, 5424–5431. doi: 10.1128/AEM.00046-12 22610437 PMC3416396

[B51] Schwieters A. Ahmer B. M. M. (2024). Identification of new sdiA regulon members of Escherichia coli, Enterobacter cloacae, and *Salmonella enterica* serovars Typhimurium and Typhi. Microbiol. Spectr. 12, e01929–e01924. doi: 10.1128/spectrum.01929-24 39436139 PMC11619404

[B52] Shankar M. Ponraj P. Illakkiam D. Rajendhran J. Gunasekaran P. (2013). Inactivation of the Transcriptional Regulator-Encoding Gene *sdiA* Enhances Rice Root Colonization and Biofilm Formation in Enterobacter cloacae GS1. J. Bacteriol. 195, 39–45. doi: 10.1128/JB.01236-12 23086212 PMC3536174

[B53] Silva-Bea S. García-Meniño I. Rey S. Romero M. Fernández J. Hammerl J. A.. (2024a). Draft genome sequence of *Klebsiella pneumoniae* KLEB-33: a convergent biofilm hyperforming multiresistant strain belonging to the emerging ST16 lineage harboring multiple hypervirulence genes. Microbiol. Resour Announc. 13, e01192–e01123. doi: 10.1128/mra.01192-23 38426732 PMC11008166

[B54] Silva-Bea S. Romero M. Parga A. Fernández J. Mora A. Otero A. (2024b). Comparative analysis of multidrug-resistant *Klebsiella pneumoniae* strains of food and human origin reveals overlapping populations. Int. J. Food Microbiol. 413, 110605. doi: 10.1016/j.ijfoodmicro.2024.110605 38308879

[B55] Smith J. N. Ahmer B. M. M. (2003). Detection of other microbial species by *Salmonella* : expression of the sdiA regulon. J. Bacteriol. 185, 1357–1366. doi: 10.1128/JB.185.4.1357-1366.2003 12562806 PMC142872

[B56] Smith J. N. Dyszel J. L. Soares J. A. Ellermeier C. D. Altier C. Lawhon S. D.. (2008). SdiA, an N-Acylhomoserine Lactone Receptor, Becomes Active during the Transit of *Salmonella enterica* through the Gastrointestinal Tract of Turtles. PLoS One 3, e2826. doi: 10.1371/journal.pone.0002826 18665275 PMC2475663

[B57] Styles M. J. Early S. A. Tucholski T. West K. H. J. Ge Y. Blackwell H. E. (2020). Chemical control of quorum sensing in E. coli : identification of small molecule modulators of sdiA and mechanistic characterization of a covalent inhibitor. ACS Infect. Dis. 6, 3092–3103. doi: 10.1021/acsinfecdis.0c00654 33124430 PMC7736514

[B58] Subramoni S. Venturi V. (2009). LuxR-family ‘solos’: bachelor sensors/regulators of signalling molecules. Microbiology 155, 1377–1385. doi: 10.1099/mic.0.026849-0 19383698

[B59] Suzuki K. Wang X. Weilbacher T. Pernestig A. K. Melefors Ö. Georgellis D.. (2002). Regulatory circuitry of the CsrA/CsrB and BarA/UvrY systems of Escherichia coli. J. Bacteriol 184, 5130–5140. doi: 10.1128/JB.184.18.5130-5140.2002 12193630 PMC135316

[B60] Tacconelli E. Carrara E. Savoldi A. Harbarth S. Mendelson M. Monnet D. L.. (2018). Discovery, research, and development of new antibiotics: the WHO priority list of antibiotic-resistant bacteria and tuberculosis. Lancet Infect. Dis. 18, 318–327. doi: 10.1016/S1473-3099(17)30753-3 29276051

[B61] Tang M. Huang Z. Zhang X. Kong J. Zhou B. Han Y.. (2023). Phage resistance formation and fitness costs of hypervirulent *Klebsiella pneumoniae* mediated by K2 capsule-specific phage and the corresponding mechanisms. Front. Microbiol. 14, 1156292. doi: 10.3389/fmicb.2023.1156292 37538841 PMC10394836

[B62] Wang H. Cai T. Weng M. Zhou J. Cao H. Zhong Z.. (2006). Conditional production of acyl-homoserine lactone-type quorum-sensing signals in clinical isolates of enterobacteria. J. Med. Microbiol. 55, 1751–1753. doi: 10.1099/jmm.0.46756-0 17108283

[B63] Wang H. Dong Y. Wang G. Xu X. Zhou G. (2016). Effect of growth media on gene expression levels in *Salmonella* Typhimurium biofilm formed on stainless steel surface. Food Control. 59, 546–552. doi: 10.1016/j.foodcont.2015.06.026

[B64] Wang S. Payne G. F. Bentley W. E. (2020). Quorum sensing communication: molecularly connecting cells, their neighbors, and even devices. Annu. Rev. Chem. Biomol Eng. 11, 447–468. doi: 10.1146/annurev-chembioeng-101519-124728 32168999

[B65] Wang Y. Wang S. Chen W. Song L. Zhang Y. Shen Z.. (2018). CRISPR-Cas9 and CRISPR-assisted cytidine deaminase enable precise and efficient genome editing in *Klebsiella pneumoniae* . Appl. Environ. Microbiol. 84, e01834–e01818. doi: 10.1128/AEM.01834-18 30217854 PMC6238054

[B66] WHO (2024). WHO Bacterial Priority Pathogens List 2024 Bacterial Pathogens of Public Health Importance, to Guide Research, Development, and Strategies to Prevent and Control Antimicrobial Resistance (Geneva: World Health Organization).

[B67] Xie Y. Wahab L. Gill J. (2018). Development and validation of a microtiter plate-based assay for determination of bacteriophage host range and virulence. Viruses 10, 189. doi: 10.3390/v10040189 29649135 PMC5923483

[B68] Yang N. Lan L. (2016). *Pseudomonas aeruginosa* Lon and ClpXP proteases: roles in linking carbon catabolite repression system with quorum-sensing system. Curr. Genet. 62, 1–6. doi: 10.1007/s00294-015-0499-5 26045103

