# Supplementary material for: Regulatory effects on virulence and phage susceptibility revealed by sdiA mutation in Klebsiella pneumoniae
Source: Front Cell Infect Microbiol. 2025 Mar 13;15:1562402. doi: 10.3389/fcimb.2025.1562402 (PMC11966055; doi:10.3389/fcimb.2025.1562402)
Supplement: Supplementary file 1 [file DataSheet1.pdf]

Supplementary Material

Regulatory effects on virulence and phage susceptibility revealed by *sdiA* mutation in *Klebsiella pneumoniae*

Sergio Silva-Bea[1], Pablo Maseda[1], Ana Otero[1**], Manuel Romero[1*]

[1]Department of Microbiology and Parasitology, Faculty of Biology - Aquatic One Health Research Center (ARCUS), Universidade de Santiago de Compostela, Santiago de Compostela, Spain.

***Correspondence:** manuelromero.bernardez@usc.es

****Co-correspondence:** anamaria.otero@usc.es

Table S1. The oligonucleotides employed in this study for the generation of *sdiA* mutants in *K. pneumoniae* are listed below.

**Element**	**Sequence**
Forward Spacer	P*5′-**TAGT**GACGTTGCCAAACCATGCTC-3′
Reverse Spacer	P*5′-**AAAC**GAGCATGGTTTGGCAACGTC-3′
ssDNA used for recombination knockout	5′-ATATTTTATACATATAAATAAGATTTATGTGTTAGTTTTCTGTATGGGCTAATCTGAATGCGCCTGCCGTTCTGCGTGTCAGACGTGATA-3′
Forward Primer	5′-CCGTATACACCGCTTCCCAT-3′
Reverse Primer	5′-CCTGTGCCGGCTGAAAGTAT-3′

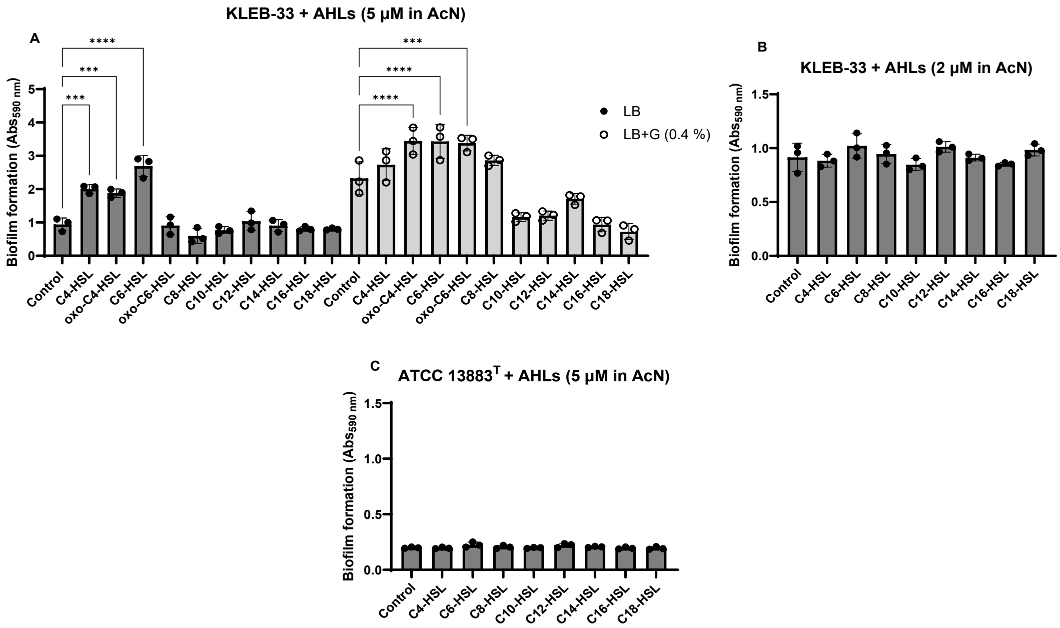

Figure S1. Effect of AHL addition on biofilm formation in *K. pneumoniae* ATCC 13883^{T} and KLEB-33 strains. The quantification of biofilm formation was conducted using CV staining, which was subsequently dissolved with 33 % acetic acid and the absorbance measured at 590 nm ($Abs_{590\ nm}$). The effect of three short-chain homoserine lactones (C4-HSL, C6-HSL and C8-HSL), two oxo- substituted short-chain homoserine lactones (oxoC4-HSL and oxo-C6-HSL) and five long-chain homoserine lactones (C10-HSL, C12-HSL, C14-HSL, C16-HSL and C18-HSL) on the AA biofilm cultivation system in the hyper-biofilm-forming strain KLEB-33 was investigated (**A**). AHLs effect was further corroborated in other experiments (**Figure 1**). No effect was observed at 2 µM in KLEB-33 strain (**B**). Neither effect was observed in the ATCC 13883^{T} strain at 5 µM (**C**) due to its inherent low biofilm-forming capacity that hindered the detection of significant differences in this biofilm model system. (N = 3). The controls have the same amount of solvent used as AHL treated samples.

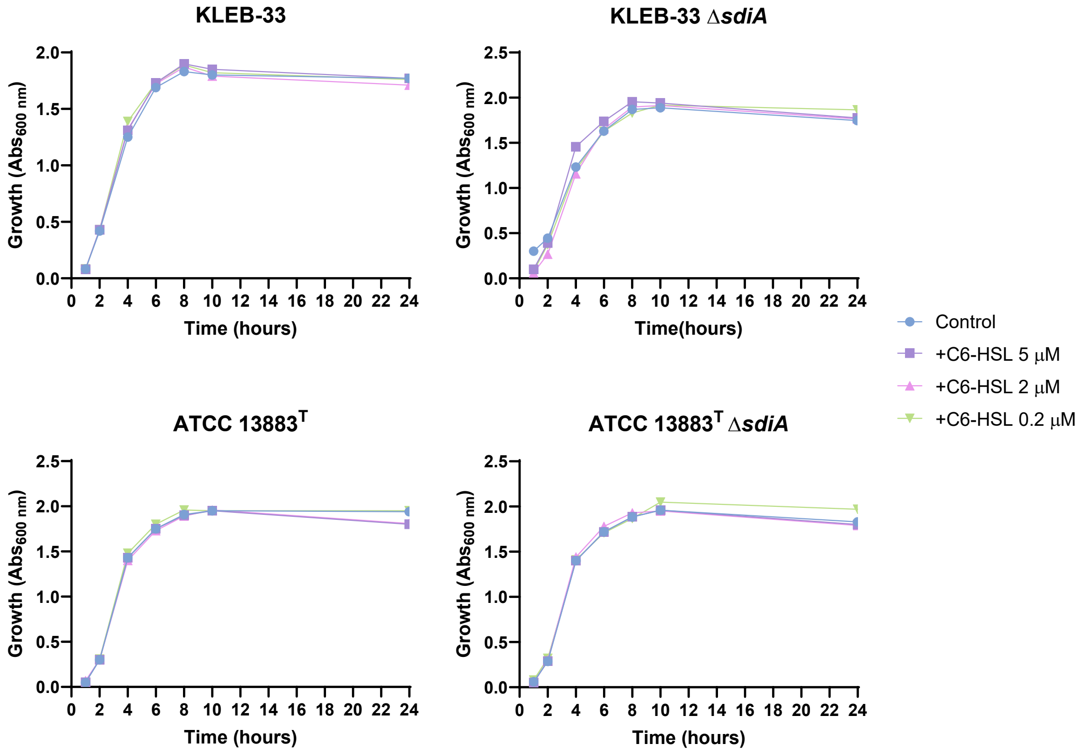

Figure S2. Growth curves of *K. pneumoniae* KLEB-33 and ATCC 13883^{T} wild-type and Δ*sdiA* strains. The impact of C6-HSL supplementation was examined at concentrations of 5, 2, and 0.2 μM. A total of three replicates per sample were used. An equal amount of solvent was added to the control cultures.

Table S2. Summary of the QQ activity results recorded from culture samples (supernatant or pellet portions) of *Klebsiella pneumoniae* KLEB-33 and ATCC 13883^T wild-type and Δ*sdiA* strains against C6-HSL (10 μM). The remaining AHL signalling activity was evaluated at 6, 12 and 24 hours of incubation. The samples were exposed to biosensor *C. subtsugae* CV026, and the presence or absence of QQ activity was recorded. This entailed observing whether the biosensor exhibited the absence of violacein production, indicative of QQ activity (+), or the presence of violacein production, indicative of negative QQ activity (-). The experiment was repeated twice (N = 3).

			Supernantant				Pellet			
	Strain	Condition	6 h	12 h	24 h	48 h	6 h	12 h	24 h	48 h
***C. subtsugae* CV026**	**ATCC 13883^T**	shaking	+	+	+	+	-	-	-	-
		static	-	+	+	+	-	-	-	-
	**ATCC 13883^T Δ*sdiA***	shaking	+	+	+	+	-	-	-	-
		static	-	-	+	+	-	-	-	-
	**KLEB-33**	shaking	+	+	+	+	-	-	-	-
		static	-	+	+	+	-	-	-	-
	**KLEB-33 Δ*sdiA***	shaking	+	+	+	+	-	-	-	-
		static	-	+	+	+	-	-	-	-

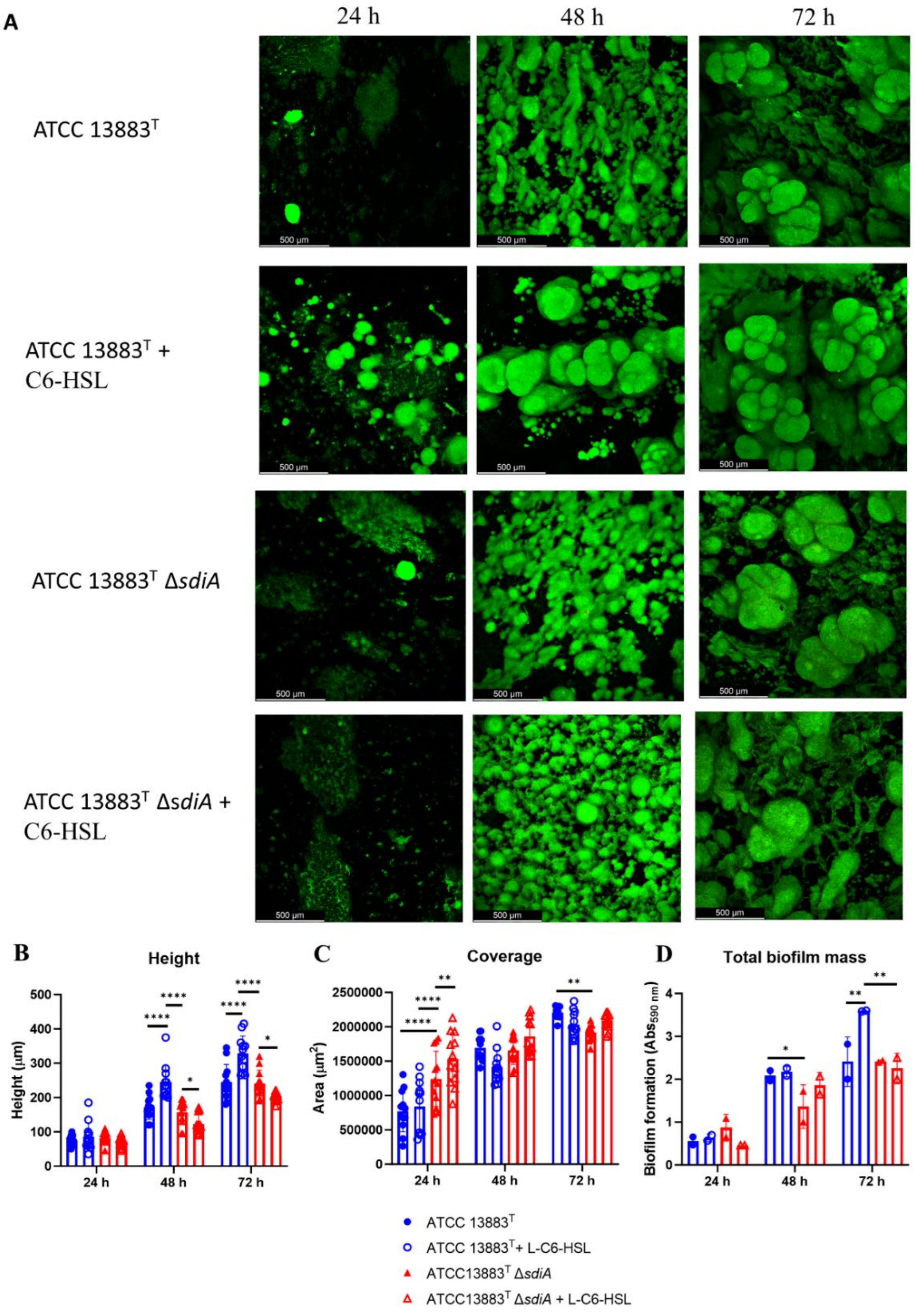

Figure S3. Impact of *sdiA* mutation and AHL addition on biofilm formation in *K. pneumoniae* ATCC 13883^T. (**A**) Representative CLSM images (N = 6) of biofilms obtained after 24, 48 and 72 h of incubation in the RBB cultivation system and staining with Syto9 fluorescent dye. (**B**) and (**C**) Quantification of height and biofilm coverage from confocal images using ImageJ (v1.54) image analysis software. (**D**) CV quantification of wild-type and Δ*sdiA* *K. pneumoniae* ATCC 13883^T strains biofilm biomass after treatment with C6-HSL (5 µM). An equal amount of solvent was added to the control cultures.

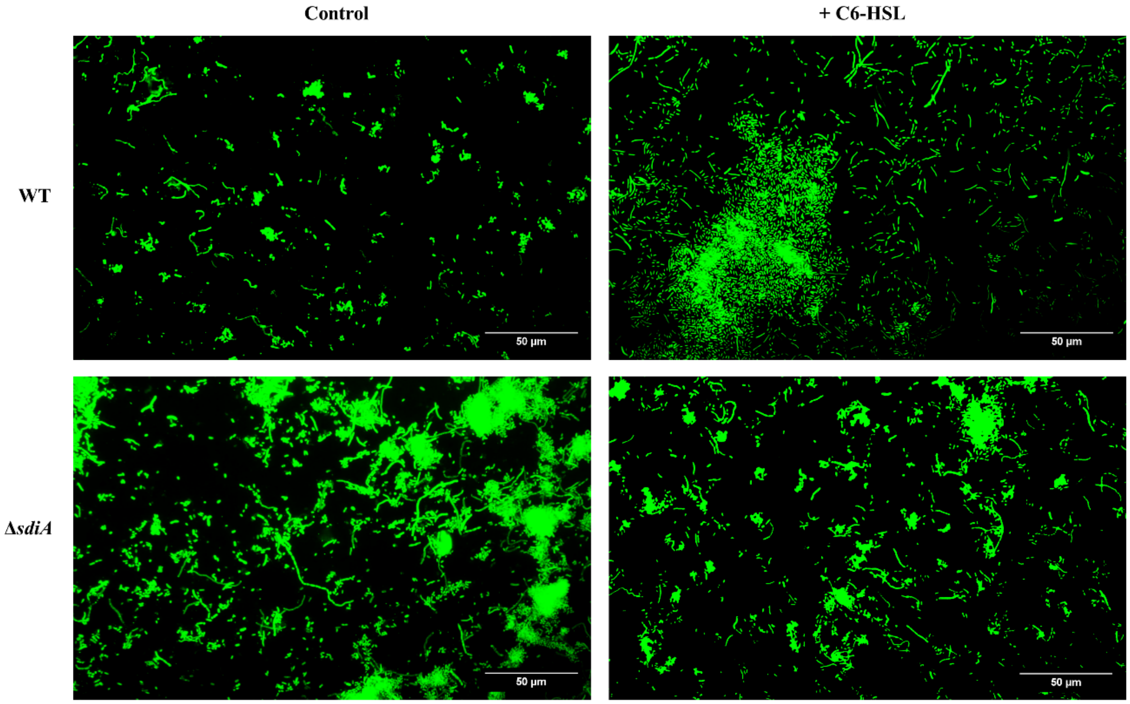

Figure S4. Representative images illustrating the presence of filamentous bacteria in the initial stages (24 h) of biofilm formation by *K. pneumoniae* KLEB-33. The biofilms were cultivated using the RBB system with or without C6-HSL (5 µM) treatment and subsequently stained with Syto9. Quantification of images is represented in **Figure 3**. An equal amount of solvent was added to the control cultures.

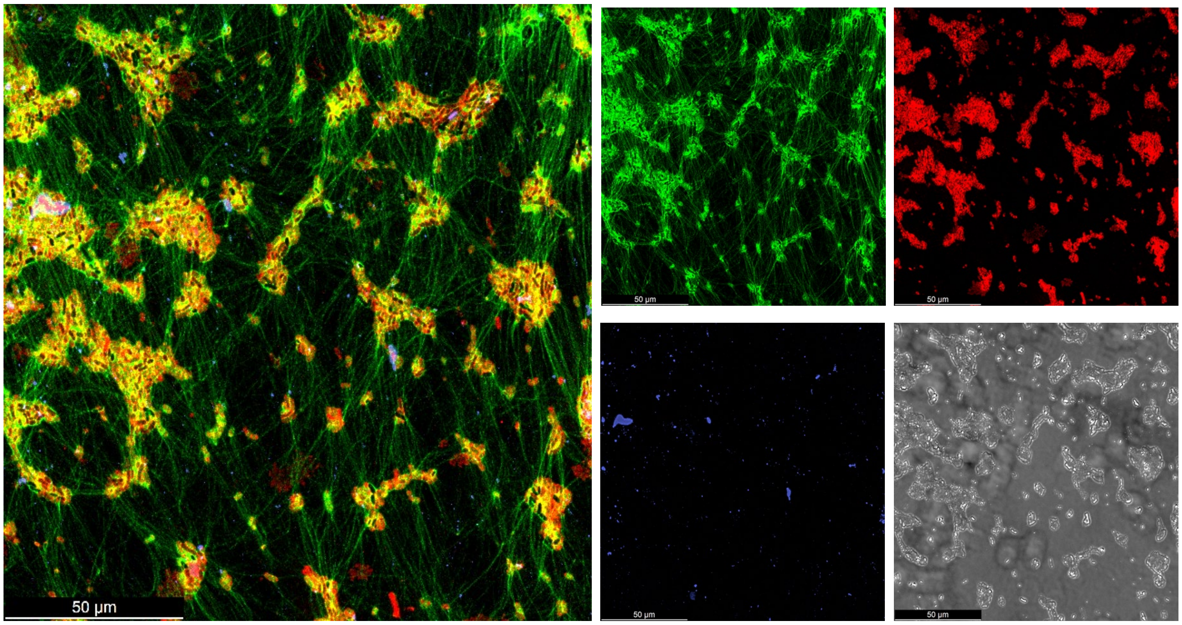

Figure S5. A representative CLSM image of the interior of mushroom-shaped structures observed in *K. pneumoniae* KLEB-33 RBB biofilms indicates that extracellular DNA (eDNA) is a primary component of these biofilms. The image pertains to a 48-hour Δ*sdiA* biofilm. The biofilms were stained with YOYO-1 (eDNA, green), FM-464 (cell membranes, red), and Concanavalin A conjugated with AlexaFluor 594 (exopolysaccharide, blue).

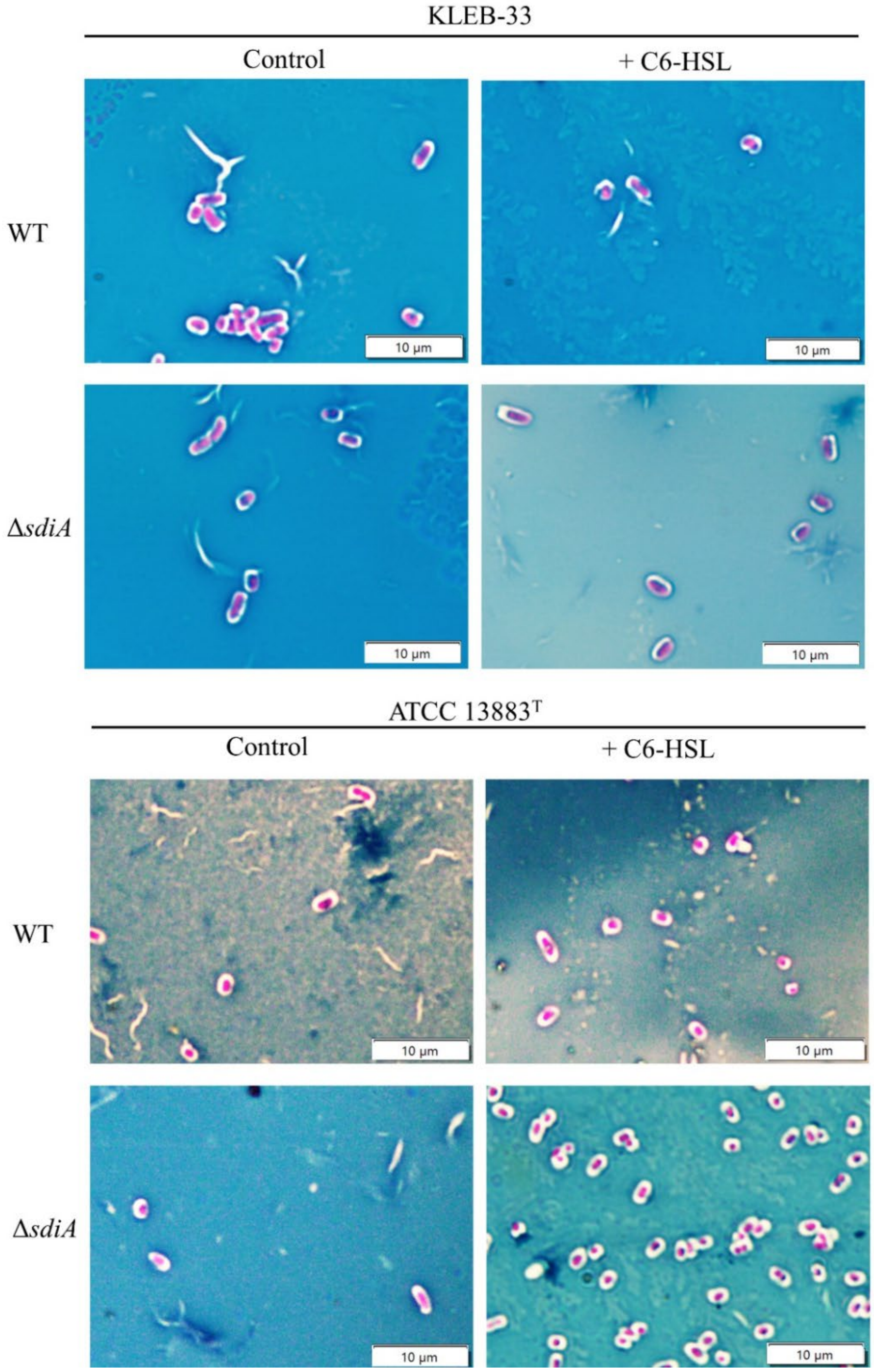

Figure S6. Representative images of Maneval capsule staining method conducted on *K. pneumoniae* KLEB-33 and ATCC 13883^{T}. The strains under consideration are wild-type (WT) and *ΔsdiA*, with and without the addition of C6-HSL (5 μM). The capsule is revealed as a clear halo between the coloured background (blue) and the stained cell (red). A total of 6 images were taken per sample with no substantial difference observed between conditions. An equal amount of solvent was added to the control cultures.

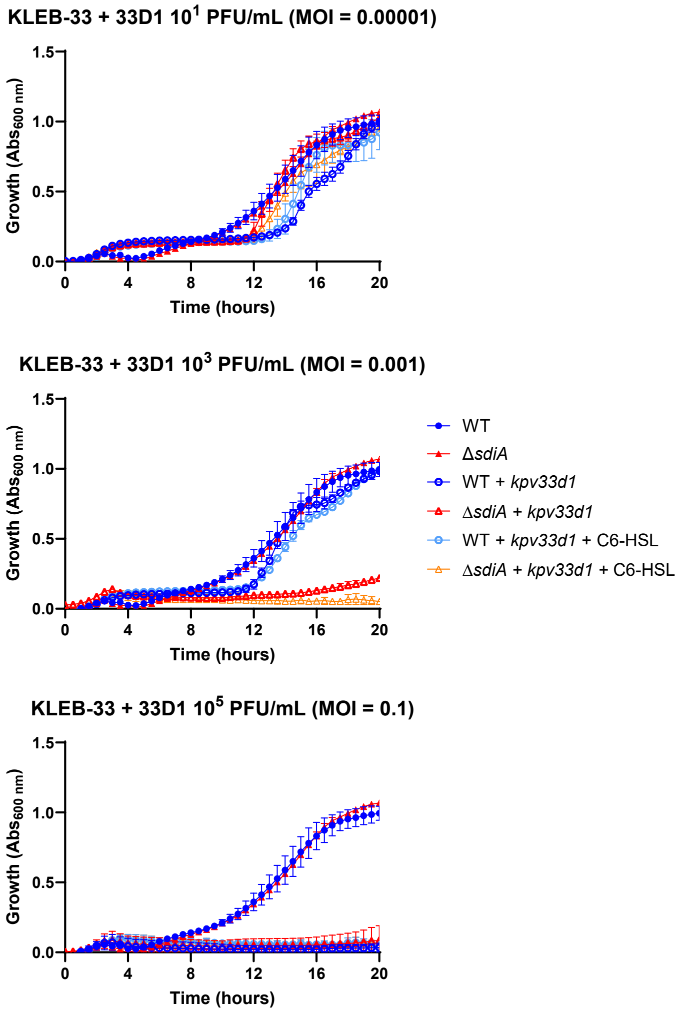

Figure S7. Susceptibility assays (N = 3) of *K. pneumoniae* KLEB-33 wild-type (WT) and Δ*sdiA* strains (10^6 CFU/mL) to *Webervirus kpv33d1* phage with C6-HSL addition (5 µM). No effect was observed regarding AHL addition. An equal amount of solvent was added to the control cultures.
